# Mechanism of exercise-derived circulating exosomes as a target for sarcopenia management

**DOI:** 10.3389/fphys.2025.1680485

**Published:** 2026-01-22

**Authors:** Xiangbo Wang, Hui Huang, Jie Chen, Qing Zhang, Zhichao Yuan, Mingyue Yin, Chenggen Peng, Songlin Liu

**Affiliations:** 1 Department of Neurosurgery, Xiangya Hospital, Central South University, Changsha, China; 2 Sport Institute, Hunan Agricultural University, Changsha, China; 3 College of Physical Education and Health, Guangxi Normal University, Guilin, Guangxi, China; 4 Physical Science College, Changsha Normal University, Changsha, China; 5 School of Athletic Performance, Shanghai University of Sport, Shanghai, China

**Keywords:** exercise, exosomes, extracellular vesicles, muscle repair, sarcopenia

## Abstract

Sarcopenia, an age-related syndrome characterized by the progressive decline of skeletal muscle mass and function, threatens the health of older adults through underlying mechanisms that include dysregulated protein metabolism, autophagy-mitochondrial dysfunction, chronic inflammation, and impaired regenerative capacity of muscle stem cells. Exercise-derived circulating exosomes, which act as key mediators of intercellular communication, show considerable potential in mitigating sarcopenia-related damage. In this review, we summarize the biogenesis of exercise-induced exosomes, encompassing both ESCRT-dependent and independent pathways, secretion regulated by RAB and SNARE proteins, and their release mediated through mechanical, calcium, metabolic, and neuroendocrine signaling during exercise. We further elaborate on the systemic roles of these exosomes in muscle repair, including alleviating lipotoxicity via the FGF21-adiponectin axis, maintaining protein homeostasis through dual regulation by miR-29c, and ameliorating the inflammatory microenvironment via modulation of macrophage polarization. Finally, we discuss the translational promise of exosomes as therapeutic targets and outline future research directions, offering a conceptual framework for understanding exercise-mediated muscle protection and developing novel interventions.

## 1 Introduction

### 1.1 Epidemiological burden of sarcopenia

Defined as age-related loss of skeletal muscle mass and function, sarcopenia leads to functional decline and loss of independence in the elderly (Cruz-Jentoft and Sayer, 2019). Sarcopenia has emerged as a critical global public health issue, severely compromising the quality of life and health status of the elderly. Accumulating epidemiological evidence indicates that the prevalence of sarcopenia increases significantly with age, particularly among individuals with chronic diseases or malnutrition (Morley et al., 2014). Beyond its adverse impacts on physical health, sarcopenia exerts profound negative effects on the quality of life of the elderly population. It is strongly associated with an increased risk of falls, fractures, hospitalization, and premature death, imposing substantial economic burdens on society and families. Additionally, sarcopenia has been linked to neurodegenerative changes such as brain atrophy, further exacerbating health issues in the elderly. Therefore, early diagnosis and intervention of sarcopenia are crucial for improving the quality of life of the elderly (Alonso-Puyo et al., 2024). However, no specific drugs have been approved for the treatment of sarcopenia to date, making the search for new therapeutic approaches a key research focus (Wen et al., 2025).

### 1.2 Basic architecture and cellular ecosystem of skeletal muscle

Skeletal muscle, one of the three major muscle tissue types in the human body, is composed of parallel bundles of muscle fibers—the fundamental contractile units. Each muscle fiber contains numerous myofibrils made of actin and myosin filaments, whose formation relies on complex processes of cellular differentiation and fusion (Feng et al., 2020). Muscle function and homeostasis are maintained by a diverse community of resident and infiltrating cells, collectively forming a dynamic cellular ecosystem or niche. The most critical resident muscle stem cells are satellite cells, which reside in a quiescent state between the basal lamina and the sarcolemma—the plasma membrane of the muscle fiber. Upon injury, satellite cells activate, proliferate, and differentiate to drive muscle growth and repair (Feng et al., 2020; Sousa-Vic et al., 2016).

Beyond these, fibroblasts and fibroadipogenic progenitors (FAPs) are responsible for producing and remodeling the extracellular matrix (ECM); in pathological conditions, they can contribute to fibrosis and fat infiltration (Plikus et al., 2021). Endothelial cells form an extensive capillary network essential for nutrient delivery, waste removal, and the provision of angiocrine signals (Rafii et al., 2016). Immune cells, such as macrophages, can adopt an anti-inflammatory, pro-regenerative phenotype to support repair, exhibiting pro-mitotic and anti-apoptotic activities that benefit the survival and growth of myogenic cells (Arnold et al., 2007). Precise communication and coordination among these cellular components are fundamental to muscle adaptation, regeneration, and the maintenance of mass and strength. Consequently, the hallmarks of sarcopenia-including protein loss, metabolic dysfunction, and regenerative failure-can be understood as a disintegration of this finely tuned architectural and cellular harmony under the pressure of aging.

This study aims to explore the potential of exercise as a non-pharmacological intervention in the prevention and treatment of sarcopenia. Exercise is known to confer clear benefits in maintaining and enhancing skeletal muscle function, partly through the regulation of various active factors, including exosomes. As key carriers of intercellular communication, exosomes demonstrate considerable regulatory potential in this context. However, several critical questions remain: How does exercise specifically promote the biosynthesis and secretion of exosomes? And how do exercise-derived exosomes act on skeletal muscle to mitigate sarcopenia-related damage and promote repair? Addressing these questions will deepen our understanding of the molecular basis of exercise-mediated muscle protection. To this end, this review will outline the pathological mechanisms of sarcopenia and synthesize recent advances in the field. It will then focus on analyzing the regulatory pathways through which exercise stimulates exosome release and will examine in detail the mechanisms by which exercise-derived exosomes contribute to injury repair in sarcopenia.

### 1.3 Pathological mechanisms of sarcopenia

#### 1.3.1 Dynamic imbalance in protein metabolic networks

A core characteristic of sarcopenia is the dynamic imbalance between muscle protein synthesis and degradation, manifested as a combined of anabolic resistance and elevated catabolic activity (Scott et al., 2011). Muscle mass loss occurs when the rate of muscle protein degradation exceeds that of synthesis, which may precede the gradual decline in muscle function (Moore, 2014). During aging, skeletal muscle exhibits a marked reduction in sensitivity to the anabolic response induced by dietary amino acids, with the molecular basis lying in the decreased activation efficiency of the mammalian target of rapamycin complex 1 (mTORC1) pathway (Drummond et al., 2012). Despite elevated phosphorylation levels of certain components of the mTORC1 signaling pathway under basal conditions, the ability of amino acid stimulation to induce mTORC1 nuclear translocation and target gene activation is impaired, leading to reduced ribosome biogenesis and synthesis of actin and myosin. Concurrently, the abnormally activated catabolic program mediated by activating transcription factor 4 (ATF4) upregulates muscle-specific ubiquitin ligases Atrogin-1 (MAFbx) and MuRF1, driving an increased degradation rate of myofibrillar proteins via the ubiquitin-proteasome system (UPS) (Moro et al., 2016).

Age-related inhibition of the insulin-like growth factor 1 (IGF-1)/PI3K/Akt/mTOR pathway further exacerbates anabolic defects. This pathway antagonizes the expression of Atrogin-1/MuRF1 by inhibiting FoxO family transcription factors (e.g., FoxO3a) (Bowen et al., 2015). However, attenuated signaling downstream of the IGF-1 receptor in aged muscle reduces Akt phosphorylation, thereby relieving the inhibition of FoxO3a and forming a vicious cycle of insufficient synthesis and excessive degradation (Wada et al., 2011). Additionally, branched-chain amino acids (BCAAs) play a crucial role in cellular metabolism, particularly in regulating protein synthesis and the mTORC1 signaling pathway. Impaired transmembrane transport efficiency of BCAAs may limit substrate supply for protein synthesis by reducing amino acid-sensing signals in mTORC1, thereby affecting cellular growth and metabolic functions (Lynch and Adams, 2014; Yoshida et al., 2019).

#### 1.3.2 Interactive dysfunction between autophagy-lysosomal system and mitochondria

Dysregulation of autophagic homeostasis is also a key node in the pathological progression of sarcopenia. In aged muscle, the expression of Beclin-1, a core molecule in the macroautophagy pathway, is increased, while the conversion efficiency of LC3-II is significantly reduced. SQSTM1/p62 plays an important role in the fusion of autophagosomes and lysosomes, accompanied by abnormal accumulation of p62/SQSTM1 protein. Its functional defects may lead to impaired degradation of autophagosomes (He et al., 2015; Salazar et al., 2020). This functional impairment reduces the clearance efficiency of damaged organelles (e.g., mitochondria) and misfolded proteins, leading to elevated levels of reactive oxygen species (ROS) in aged muscle cells, which in turn induce oxidative stress and further inhibit anabolism by oxidatively modifying mTORC1 complex subunits (Goode et al., 2016). Mitochondrial dysfunction and autophagic impairment form a positive feedback loop. A recent study demonstrated that mitochondrial protein homeostasis and fission are altered in aged muscle. Fission and mitophagy are shown to increase with age, while mitochondrial content decreases in both slow and fast muscle fiber types (Murgia et al., 2017).

Mitochondria in aged skeletal muscle exhibit dynamic imbalance, with decreased transcriptional activation efficiency of the mitochondrial biogenesis pathway (PGC-1α/NRF1/TFAM axis), leading to reduced mitochondrial DNA copy number and exacerbating energy metabolism defects, a phenomenon confirmed in various pathological states. Studies have shown that PGC-1α is a key regulator of mitochondrial biogenesis, and its downstream target genes include nuclear respiratory factor 1 (NRF1) and mitochondrial transcription factor A (TFAM), which collectively participate in the regulation of mitochondrial DNA replication and transcription (Shen et al., 2014; Chen et al., 2018). Impaired selective clearance capacity of mitophagy, characterized by reduced Parkin-mediated ubiquitination of mitochondria, results in the persistent accumulation of dysfunctional mitochondria. The caspase-3-dependent apoptotic pathway promotes apoptotic loss of muscle fibers, which is a key determinant of age-related muscle loss (Kob et al., 2015).

#### 1.3.3 Chronic inflammation

Chronic low-grade inflammation is recognized as a pivotal driver in the pathogenesis and progression of sarcopenia. Elevated levels of inflammatory factors can promote muscle protein degradation and inhibit protein synthesis (Ogawa et al., 2016). In cancer-related cachexia, inflammatory factors such as IL-6 and TNF-α affect muscle protein metabolism through multiple pathways, leading to muscle atrophy (Feng et al., 2025). Inflammatory responses are the primary mediators of these metabolic alterations, and interventions targeting inflammation may help alleviate the net catabolic effect on muscle protein metabolism (Durham et al., 2009). Furthermore, chronic inflammation promotes muscle atrophy by influencing muscle autophagy and the ubiquitin-proteasome system (UPS), which further emphasizes the central role of inflammation in sarcopenia (Webster et al., 2020). Chronic inflammation can activate pro-inflammatory pathways, such as the NF-κB pathway, which can promote muscle atrophy and adipose tissue expansion. A study on the impact of inflammation on cancer demonstrated that chronic inflammation can induce immune suppression, creating a favorable environment for carcinogenesis (Akkız et al., 2025). Similarly, in sarcopenia, chronic low-grade inflammation may contribute to the development of muscle damage and adipose tissue dysfunction. Another important aspect is the role of hormonal changes. Age-related alterations in the hormonal environment, such as decreased levels of testosterone and growth hormone, can lead to muscle loss and increased adipose tissue accumulation. Insulin resistance, commonly associated with obesity, can disrupt the normal regulation of muscle metabolism, contributing to muscle atrophy. Insufficient nutrient intake, metabolic disorders, hormonal imbalances, and mitochondrial dysfunction are also involved in this process.

#### 1.3.4 Age-related muscle stem cell dysfunction and niche disruption

During the aging process, the homeostasis and function of muscle stem cells (MuSCs) progressively decline. While the total number of MuSCs may decrease with age, a more pivotal change lies in their functional state. Research indicates that aged MuSCs exhibit a diminished capacity for activation, proliferation, and differentiation, which directly undermines the muscle’s ability to repair (Cai et al., 2022; Snijders and Parise, 2017; Fry et al., 2015). These intrinsic deficits include metabolic shifts, altered polarity, and epigenetic modifications. For instance, age-related methylation changes can silence genes essential for stem cell self-renewal and quiescence, thereby depleting the regenerative reserve and contributing to sarcopenia progression (Bigot et al., 2015; Chinvattanachot et al., 2024).

Aging induces a profound dysregulation of the overall architecture and signaling networks within the niche. The niche, composed of muscle fibers, endothelial cells, fibroblasts, and immune cells, relies on precise regulation to maintain its function (Li W. et al., 2025). However, a combination of chronic low-grade inflammation, dysregulated extracellular matrix (ECM) composition, impaired vascular support, and a pro-fibrotic shift collectively fosters an inhibitory microenvironment that compromises MuSC function and disrupts intercellular communication (Sousa-Vic et al., 2016; Guillon et al., 2025). Inflammatory cytokines such as IL-1α, IL-13, TNF-α, and IFN-γ present within the inflammatory milieu have been found to stimulate the proliferation of muscle stem cells (MuSCs) and promote their expansion *in vitro* (Fu et al., 2015). This cytokine combination, mediated by inflammation, can rapidly activate MuSCs following muscle injury, promoting their proliferation and differentiation, thereby accelerating the repair process. However, under conditions of chronic inflammation, immune cells such as macrophages may express high levels of transforming growth factor-β1 (TGF-β1). This can inhibit the apoptosis of fibro/adipogenic progenitors, driving their differentiation into matrix-producing cells and consequently promoting fibrosis (Lemos et al., 2015).

Within the aged muscle stem cell microenvironment, increased expression of fibroblast growth factor 2 (Fgf2) leads to the loss of quiescence and self-renewal capacity in a subset of satellite cells (Chakkalakal et al., 2012). Furthermore, intrinsic alterations occur in muscle stem cells during aging, such as increased activity of the p38α and p38β mitogen-activated protein kinase pathways, which further impair their regenerative potential (Cosgrove et al., 2014). Therefore, the impaired regenerative output in sarcopenia is best understood as the downstream result of a degenerative cycle: aging erodes the functional competence of MuSCs and corrupts their regulatory niche, which together lead to a failure in mounting effective repair responses.

The pathological mechanisms of sarcopenia form a multi-layered and intricately interconnected network, with its core lying in the systemic homeostatic imbalance induced by aging. This imbalance is specifically manifested as: a dynamic imbalance in the protein metabolic network; dysfunction of the autophagy-mitochondrial axis, where impaired autophagic flux and decreased mitochondrial biogenesis create a vicious cycle; chronic low-grade inflammation, which promotes protein degradation and inhibits synthesis; and the functional exhaustion of muscle stem cells coupled with microenvironment dysregulation. These mechanisms do not exist in isolation but rather interact and influence each other, collectively forming a vicious cycle characterized by reduced synthesis, increased breakdown, failed clearance, and ineffective repair. Ultimately, this leads to the progressive loss of skeletal muscle mass and function.

## 2 Biogenesis of exercise-induced circulating exosomes

### 2.1 Basic characteristics of exosomes

Exosomes are nanoscale extracellular vesicles secreted by virtually all cell types, possessing unique biological properties. They typically have a diameter ranging from 30 to 140 nm and a lipid bilayer membrane structure, and are a key subclass of extracellular vesicles (Safdar and Tarnopolsky, 2018). Extracellular vesicles are small membrane-enclosed vesicles that originate from multivesicular bodies or the plasma membrane. Virtually all cell types release extracellular vesicles, which are present in a wide range of bodily fluids such as blood and milk. Their biogenesis occurs primarily through two pathways: one involves direct outward budding or shedding of the plasma membrane to generate larger microvesicles, while the other entails inward budding of the membrane of intracellular multivesicular bodies to form intraluminal vesicles; subsequent fusion of these multivesicular bodies with the plasma membrane releases the intraluminal vesicles into the extracellular space as nanoscale exosomes (Pegtel and Gould, 2019; Robbins and Morelli, 2014).

Their molecular composition is highly complex and functionally diverse, comprising three major classes of biomolecules: lipids, proteins, and nucleic acids (Isaac et al., 2021). Lipids are characterized by phosphatidylserine enriched in the outer membrane, cholesterol that maintains membrane stability, sphingomyelin involved in signal transduction, and ceramide that drives ESCRT-independent biogenesis (Nail et al., 2023). Proteins include structural proteins (e.g., cytoskeletal proteins, membrane - binding proteins); functional proteins (HSP70/HSP90, Rab5/7, integrins); signature proteins (Tetraspanins CD63/CD81/CD9), and transferrin receptor (Fan et al., 2023). Nucleic acids carry genetic information substances such as mitochondrial DNA (mtDNA), translatable mRNAs, and regulatory non-coding RNAs (ncRNAs, lncRNAs, and circRNAs) (Jung et al., 2025).

**FIGURE 1:**
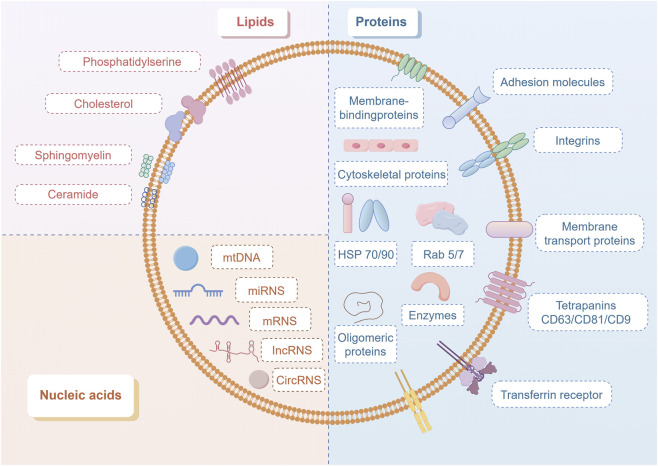
Schematic diagram of exosomal molecular composition.

Exosomes and their biogenesis process play an important role in maintaining protein quality, as their release can trigger the reorganization of the surrounding extracellular matrix and facilitate intercellular communication. Once secreted, exosomes can enter the interstitial space and eventually the circulatory system, exerting effects in local paracrine or distal systemic pathways. They are key components of intercellular and interorgan communication systems, capable of carrying biological signals from 1 cell type or tissue to another (Jørgensen et al., 2013). In metabolic processes, exosomes regulate metabolic pathways in target cells by transferring bioactive molecules such as miRNAs, participating in the pathogenesis and progression of metabolic diseases (Cunha et al., 2024) (Figure 1, by figdraw.com).

### 2.2 Molecular mechanisms of exosome biogenesis

The biogenesis of exosomes proceeds through a highly regulated cascade, encompassing the invagination of the plasma membrane to form early endosomes, the invagination of multivesicular bodies (MVB) to generate intraluminal vesicles (ILV), and the fusion of MVB with the plasma membrane for exosome release. Its molecular mechanisms involve two core pathways: ESCRT-dependent and ESCRT-independent (Wei et al., 2021; Yanagawa et al., 2024). Exosome biogenesis initiates with the invagination of the plasma membrane to form endosomes, and the endosomal membrane further buds inward to generate MVB containing ILV. In the ESCRT-dependent pathway, the classical regulatory mechanism relies on ESCRT complexes (ESCRT-0, -I, -II, -III) coordinated with ubiquitination-based sorting mechanisms. These complexes mediate the recognition and enrichment of membrane-bound cargo, and drive the invagination of the MVB membrane. ESCRT complexes play a pivotal role in MVB biogenesis: ESCRT-0 is responsible for recognizing and clustering ubiquitinated cargo, ESCRT-I and ESCRT-II function in the inward budding of the membrane, and ESCRT-III mediates membrane scission to complete ILV formation. These vesicles then transport the cargo to lysosomes for degradation (Wollert and Hurley, 2010). In contrast, the ESCRT-independent pathway relies on adaptor molecules such as tetraspanins and Syntenin-ALIX, and mediates the invagination of the MVB membrane to form ILV through the synergistic action of lipid microdomains and protein complexes (Bissig and Gruenberg, 2014). The two pathways differ in their focus on cargo sorting and the regulation of membrane dynamics (Colombo et al., 2014).

MVB have two fates after formation: some fuse with lysosomes, and their contents are degraded; others proceed to the secretory pathway, and ILV are released into the extracellular space through fusion with the plasma membrane. If these released ILVs also meet the established size criteria for exosomes (30–140 nm), they are defined as exosomes; otherwise, they are more broadly classified as EVs. ILV are the precursor structures of exosomes, and they are enriched with bioactive molecules (such as RNA and proteins) derived from donor cells. Moreover, the composition of these components differs significantly from that of the parent cells, which stems from the specific cargo sorting mechanism during MVB formation (Kalluri and LeBleu, 2020).

**FIGURE 2:**
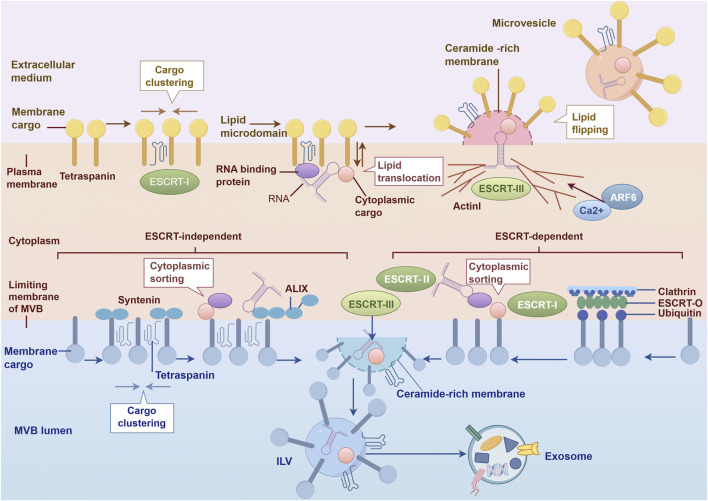
Schematic diagram of the molecular mechanism of exosome biogenesis.

Exosomes stably contain characteristic markers such as tetraspanins (CD9, CD63, CD81), heat shock proteins (HSP60, HSP70), and ESCRT-related components (Alix, TSG101) (Théry et al., 2002; Regimbeau et al., 2022; Simons and Raposo, 2009; Li W. et al., 2017), providing a molecular basis for their identification. The secretion process of exosomes mainly depends on the synergistic action of RAB family small GTPases and SNARE family proteins. RAB proteins (e.g., RAB11, RAB35, RAB27A/B) regulate the transport, localization, and docking of MVB with the plasma membrane through interaction with the cytoskeleton (Savina et al., 2005; H et al., 2010). SNARE complexes (e.g., VAMP7, YKT6, etc.) promote the fusion of MVB with the plasma membrane and the final release of exosomes by mediating membrane fusion (Gross et al., 2012; Zylbersztejn and Galli, 2011) (Figure 2).

### 2.3 Exercise-induced biogenesis of exosomes

Exercise can induce the rapid release of exosomes. Evidence indicates that in healthy individuals, after exhaustive exercise such as cycling or running, exercise significantly increases nanoscale exosomes in the blood, and exosome release begins early in exercise, even before the individual reaches the anaerobic threshold. In a study involving healthy individuals undergoing incremental cycling exercise, it was found that small extracellular vesicles with exosome-characteristic sizes and carrying exosome-specific proteins in plasma increased significantly immediately after exercise, and returned to pre-exercise levels after 90 min of rest (Frühbeis et al., 2015). In a study involving nine healthy young men, venous blood samples were collected before, during, and throughout the recovery period of a 1-h moderate-intensity (or high-intensity) semi-recumbent cycling exercise and a time-matched rest control trial. Platelet-derived exosomes increased from baseline only during high-intensity exercise. During exercise, platelet microvesicles (PMV) were correlated with brachial artery shear rate and plasma norepinephrine concentration; compared with resting exosomes, exercise-derived exosomes enhanced endothelial cell proliferation, migration, and tube formation (Wilhelm et al., 2016). These results indicate that high-intensity exercise leads to a substantial increase in circulating PMV, which may play a role in exercise-mediated vascular healing and adaptation. As a type of extracellular vesicle, exosomes play a key role in intercellular communication, serving as important carriers for intercellular information transmission and capable of regulating cellular physiological functions and phenotypes (Zylbersztejn and Galli, 2011).

Exercise-induced circulating exosomes have a complex and diverse composition, containing multiple biomolecules that play important roles in intercellular communication and physiological regulation. Proteins are key components of exosomes; exosomes from different sources have specific protein profiles involved in various cellular physiological processes such as signal transduction and metabolic regulation. In plasma exosomes from patients with primary biliary cirrhosis, proteins related to immune regulation can be detected, which can modulate the expression of co-stimulatory molecules in monocyte populations and influence immune responses (Tomiyama et al., 2017). Exosomes trigger biological changes in recipient cells by delivering their carried bioactive substances such as proteins and nucleic acids. In metabolic diseases, exosomes secreted by adipocytes can carry miRNAs related to lipid metabolism and transfer them to liver or skeletal muscle cells, regulating lipid metabolism processes in these cells (Li W. et al., 2017).

Exosomes are rich in nucleic acids, including miRNAs, mRNAs, and mitochondrial DNA. miRNAs are particularly important in exercise-induced circulating exosomes, as they can regulate target gene expression and participate in cellular processes such as proliferation, differentiation, and apoptosis. Studies have found that the expression levels of certain miRNAs in circulating exosomes change significantly after exercise, such as miR-21 and miR-26, which may be related to exercise-induced physiological adaptation and tissue repair (Karvinen et al., 2020). Multiple muscle-related microRNAs (miRNAs) are upregulated in circulating exosomes after exercise, including but not limited to miR-1, miR-133a/b, miR-206, miR-208a, and miR-499 (Castaño et al., 2020; Vechetti et al., 2021; Annibalini et al., 2019; D'Souza et al., 2018). These changes have been reported after both short-term and long-term exercise interventions. Importantly, various exercise modalities have been confirmed to induce an increase in the total number of exosomes in the blood, especially subpopulations of exosomes enriched with miRNAs (Gomes et al., 2014; Kawanishi et al., 2023). Although this research field is relatively new, studies have explored the impact of acute exercise on specific exosome subtypes through detailed analysis of exosomal surface markers and their carried gene expression profiles, revealing that exercise significantly regulates the expression of exosomal surface markers. Exosomes are also involved in immune regulation and inflammatory responses. In immune responses, exosomes secreted by immune cells can regulate the activity of other immune cells, influencing the intensity and direction of immune responses: exosomes from dendritic cells can activate T cells to enhance immune responses, while exosomes from regulatory T cells have immunosuppressive effects, inhibiting excessive immune reactions (Agarwal et al., 2014).

Exercise triggers the release of circulating exosomes through multi-level physiological stimuli, with core mechanisms involving the synergistic action of mechanical signal transduction, calcium ion oscillations, energy stress responses, and neuroendocrine regulation (Whitham et al., 2018). These pathways interact synergistically to regulate exosome biogenesis and secretion, with calcium signaling acting as a central hub. The rapid release of exosomes during exercise may also be triggered by multiple exercise-related physical and biochemical signals. Initial stimuli may originate from direct mechanical signals such as blood flow shear stress and muscle contraction itself (Hou et al., 2019; Obi et al., 2025). Exercise may regulate the formation and secretion of exosomes by affecting intracellular calcium ion concentration and protein kinase activity, and may also influence intracellular membrane transport systems, promoting the fusion of multivesicular bodies with the cell membrane to release exosomes into the extracellular environment (Vechetti et al., 2021). In skeletal muscle, action potentials induced by motor neurons trigger the massive release of Ca^2+^ from the sarcoplasmic reticulum; these Ca^2+^ not only participate in excitation-contraction coupling but may also promote exosome release more rapidly than in other tissues (Whitlock and Hartzell, 2017). Exercise triggers intracellular Ca^2+^ oscillations through mechanical stress (e.g., Piezo1 activation) and metabolic stress (AMPK/mTORC1 pathway) (Zhang et al., 2021), which in turn activate calmodulin and SNARE complexes (Zhang et al., 2021; Amemiya et al., 2021), ultimately driving MVB anchoring and fusion with the plasma membrane (Ohya et al., 2024). When plasma membrane receptors are activated, integrins sense deformation of the extracellular matrix and promote endosome formation through the FAK/PI3K signaling pathway; meanwhile, the Piezo1 ion channel responds to membrane changes, mediating Ca^2+^ influx and driving MVB transport (Li et al., 2014). Ca^2+^ plays a core regulatory role in exercise-promoted exosome release: during exercise, motor neurons generate action potentials, prompting the opening of sarcoplasmic reticulum RyR channels and a transient increase in cytoplasmic Ca^2+^ concentration (Savina et al., 2003); Ca^2+^ activates Rab GTPases (Rab27a/Rab35) through calmodulin, promoting MVB anchoring to the plasma membrane (Ostrowski et al., 2010); Synaptotagmin-7 mediates the assembly of SNARE complexes (VAMP7/STX4) to initiate membrane fusion (Fader et al., 2009).

**FIGURE 3:**
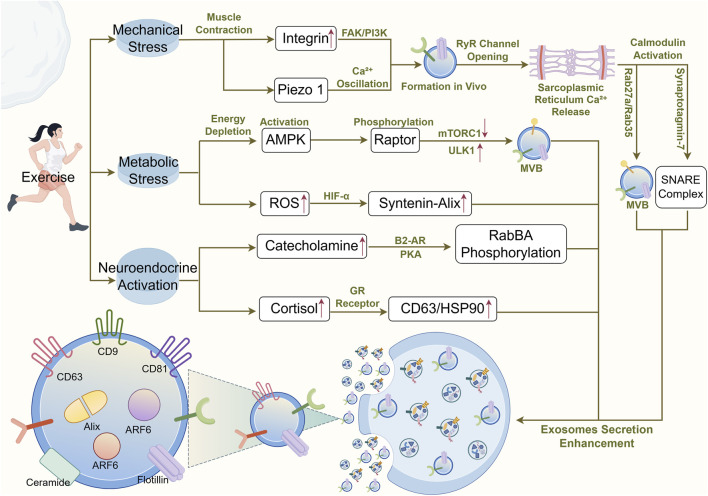
Schematic diagram of the mechanism underlying exercise-induced exosome secretion.

Energy consumption during exercise can promote AMPK activation, during which phosphorylated Raptor inhibits mTORC1, thereby relieving inhibition of ULK1 and activating autophagy-related MVB formation (Saikia and Joseph, 2021). Reactive oxygen species (ROS) also increase transiently, promoting HIF-1α stabilization and upregulating the Syntenin-Alix pathway, which regulates exosome biogenesis and release through an ESCRT-independent pathway (King et al., 2012). Concurrently with elevated ROS, catecholamine hormones (epinephrine and norepinephrine) increase, activating the PKA signaling pathway through β2-AR receptors and phosphorylating Rab8A to enhance exosome secretion (Singh and Moniri, 2012; Rambacher and Moniri, 2020). Increased cortisol promotes GR receptor-mediated upregulation of CD63 and HSP90 expression, facilitating exosome release (Whitham et al., 2018) (Figure 3).

Regarding the cellular sources of exercise-induced exosomes, existing evidence indicates their heterogeneity. Flow cytometry or immunocapture analyses have shown that these exosomes are primarily derived from lymphocytes (CD4^+^, CD8^+^), monocytes (CD14^+^), endothelial cells (CD105^+^, CD146^+^), and platelets (CD41^+^) (Brahmer et al., 2019; Bocchetti et al., 2024; Burrello et al., 2022) (Table 1). Skeletal muscle tissue has also been identified as a contributor, which can be characterized by the specific marker α-sarcoglycan (SGCA^+^). Even low-intensity exercise is sufficient to stimulate exosome secretion, and the release of exosomes tends to increase with the prolongation of exercise duration or the enhancement of exercise intensity (Gomes et al., 2017). The release intensity of skeletal muscle-specific exosomes (SGCA^+^) is positively correlated with exercise duration and intensity (Hoffman et al., 2025). However, the exact function of skeletal muscle-derived exosomes in exercise physiological responses remains insufficiently understood. Although skeletal muscle is known to release various factors (such as myokines) during exercise, its relative contribution to the total pool of circulating exosomes observed after exercise has not been clarified. Current viewpoints tend to suggest that exosome release during exercise is the result of multiple cell types (immune cells, endothelial cells, platelets, and muscle cells) collectively responding to exercise stimuli.

**TABLE 1:** Cellular sources and functional characteristics of exercise-induced exosomes.

Cellular source	Key molecular markers	Core biological functions	Regulatory mechanisms
Platelets	CD41^+^, P-selectin	Promote angiogenesis, enhance endothelial repair, and improve endothelial cell proliferation, migration, and tube formation capacity	Respond to blood flow shear stress, activate the FAK/PI3K pathway via β-integrins; catecholamine-PKA-Rab8A axis promotes release (Wilhelm et al., 2016; Zhang et al., 2021)
Skeletal muscle cells	SGCA^+^, α-sarcoglycan, miR-206	Facilitate muscle regeneration and repair, activate satellite cells, and inhibit MuRF1/Atrogin-1 expression	Muscle contraction induces Ca^2+^-PKC pathway activation; ROS-HIF-1α upregulates the Syntenin-Alix pathway (King et al., 2012)
Endothelial cells	CD146^+^, eNOS	Maintain vascular homeostasis, improve endothelial function, and enhance NO-mediated vasodilation and anti-inflammatory effects	Laminar shear stress activates the Piezo1-Ca^2+^ channel; AMPK/mTORC1-ULK1 pathway drives MVB formation (Whitham et al., 2018; Zhang et al., 2021; Saikia and Joseph, 2021)
Immune cells	CD14^+^, CD4^+^, CD8^+^	Regulate the inflammatory microenvironment, promote M2 macrophage polarization, and inhibit excessive immune responses	Cortisol-GR receptor upregulates CD63/HSP90; catecholamines enhance exosomal immunomodulatory functions via β_2_-AR (Agarwal et al., 2014; Whitham et al., 2018)
Other cells	HSP70/90, Tetraspanins	Systemic metabolic regulation, modulate lipid oxidation, and improve insulin sensitivity	Synergistic action of metabolic stress-induced AMPK activation, Ca^2+^, and the Synaptotagmin-7-SNARE axis (Amemiya et al., 2021; Ohya et al., 2024; Saikia and Joseph, 2021)

Abbreviations: *SGCA* ^*+*^, α-sarcoglycan positive; *PKA*, Protein Kinase A; *Rab8A*, Ras-related protein Rab-8A; *Ca* ^*2+*^ *-PKC*, Calcium Ion-Protein Kinase C; *ROS*, reactive oxygen species; *HIF-1α*, Hypoxia-Inducible Factor 1α; *NO*, nitric oxide; *GR*, glucocorticoid receptor; *HSP90*, Heat Shock Protein 90; *β*_*2*_ *-AR*, β_2_-Adrenergic Receptor; *HSP70*, Heat Shock Protein 70.

While exercise is a potent stimulus for exosome release, determining the precise tissue of origin for circulating exosomes remains a significant challenge. Although skeletal muscle is a major secretory organ during physical activity, the systemic nature of exercise involves simultaneous metabolic flux in the liver, adipose tissue, endothelial cells, and the immune system (Fan et al., 2021; Saçma and Geiger, 2021). Consequently, the exercise-induced exosome pool is highly heterogeneous, likely containing vesicles from all these tissues rather than muscle alone. Furthermore, the fate of these vesicles—whether they are 'targeted’ to specific acceptor tissues via surface ligand-receptor interactions or simply distributed stochastically via circulation remains under investigation. Recent evidence supports a degree of organotropism, such as the preferential uptake of muscle-derived exosomes by the liver, yet, likely, a substantial portion of these vesicles are widely distributed or cleared by the reticuloendothelial system (Whitham et al., 2018). Thus, exercise-induced exosomes should be viewed as a systemic signaling network rather than a purely myokine-like phenomenon.

## 3 Systemic roles of exercise-induced circulating exosomes in muscle repair

### 3.1 Alleviating lipotoxicity

Exosomes play a significant role in mitigating lipotoxicity, thereby helping to maintain normal myocyte function. Lipotoxicity refers to the phenomenon in which excessive intracellular lipid accumulation leads to cellular dysfunction and damage. In the context of sarcopenia, age-related metabolic disorders often cause ectopic lipid deposition in skeletal muscle, which significantly impairs metabolic function, exacerbates insulin resistance, and promotes muscle mass loss (Bosma et al., 2012; Borén et al., 2013). In individuals with obesity and type 2 diabetes, lipotoxicity not only contributes to insulin resistance but may also directly drive muscle atrophy (Meex et al., 2019). Therefore, targeting lipotoxicity represents an important strategy for delaying the progression of sarcopenia.

Research demonstrates that exosomes derived from mesenchymal stem cells can regulate lipid metabolism by activating the FGF21-adiponectin axis: exosomes inhibit skeletal muscle lipoprotein lipase (LPL) activity via the ANGPTL4 protein, while simultaneously activating the hepatic miR-122-5p/Keap1/Nrf2 axis to promote FGF21 synthesis, thereby driving systemic fatty acid oxidation (accompanied by increased CPT1a expression) (Kim et al., 2024). This regulatory mechanism alleviates high-fat-diet-induced hepatic steatosis and insulin resistance in obese mice, consequently reducing lipid-mediated toxicity to muscle cells (Kim et al., 2024). From the perspective of sarcopenia, activation of this pathway may help improve metabolic flexibility in aged muscle, attenuating the negative impact of lipid accumulation on contractile function and protein synthesis.

Specifically, exosomes may alleviate lipotoxicity through multiple mechanisms. On one hand, exosomes can modulate the expression of lipid-metabolism-related genes, promoting fatty acid oxidation and reducing intracellular lipid accumulation. For example, in primary grass carp hepatocytes, exosomes derived from fatty hepatocytes enrich glucose-regulated protein 78 (GRP78) to activate inositol-requiring enzyme-1α (IRE1α), thereby promoting lipid accumulation; conversely, inhibiting the GRP78 or IRE1α pathway reduces lipid accumulation, highlighting the regulatory role of exosomes in lipid metabolism (Yang et al., 2024). On the other hand, exosomes can mitigate inflammation-induced damage caused by lipotoxicity by modulating inflammatory responses. For instance, macrophage-derived exosomes protect bone in an osteoporotic mouse model by delivering miR-3102-5p, which suppresses lipid peroxidation and inflammatory responses, thereby indirectly alleviating the impact of lipotoxicity on the musculoskeletal system (Geng et al., 2024) (Table 2). These anti-inflammatory and metabolic regulatory effects suggest that exercise-derived exosomes may, through similar pathways, counteract chronic low-grade inflammation and disordered lipid metabolism in aging individuals, thereby protecting muscle from lipotoxic damage.

**TABLE 2:** Effects of exercise on exosome biogenesis.

Author	Experimental model	Age	Intervention protocol	Model type	Sample type	Post-exercise outcomes	Core molecular mechanisms
Whitham et al. (2018)	Healthy humans	27 ± 1 years old	1-h acute cycling exercise	Human trial	Circulating EV	Increased release of over 300 EV-associated proteins	EV-carried proteins accumulate in the liver, drive inter-tissue communication, and activate fatty acid oxidation
Kim et al. (2024)	High-fat diet-induced obese mice	19 weeks old	Mesenchymal stem cell-derived exosome intervention	Animal model	Liver/skeletal muscle	Reduced hepatic steatosis, improved insulin sensitivity	Activated FGF21-adiponectin axis; ANGPTL4 reduced LPL activity by 72%; miR-122-5p/Keap1/Nrf2 increased FGF21 (3.8-fold); enhanced fatty acid oxidation (CPT1a upregulation)
Sullivan et al. (2022)	Obese mice	18–35 weeks old	7-day combined aerobic + resistance exercise	Animal model	Skeletal muscle small EV	Altered miRNA profiles targeting inflammation/growth pathways	Regulated Wnt/β-catenin, PI3K/AKT, IGF-1, PTEN, PEDF pathways; reduced Jun, Fos, and IL-8 mRNA
Oliveira et al. (2018)	Healthy rats	Not reported	Acute aerobic exercise	Animal model	Serum EV	Changes in EV diameter, concentration, and small RNA content	Exercise intensity-dependent mechanism of EV release
Lovett et al. (2018)	Muscle injury model	18–30 years old	Two bouts of muscle-damaging exercise	Human trial	Circulating EV	Dynamic changes in muscle-specific miRNAs	miR-206, miR-133, etc., involved in muscle repair
Barone et al. (2016)	Healthy rats	Not reported	Endurance training	Animal model	Skeletal muscle tissue	Increased expression of heat shock protein HSP60	HSP60 induced PPARγ coactivator expression, promoting metabolic adaptation
Rigamonti et al. (2020)	Healthy obese population	12.4–36.5 years old	Acute exercise	Human trial	Circulating EV	EV count regulated by BMI and gender	Mechanisms underlying tissue-specific differences in EV release
Wang et al. (2019)	Muscle atrophy model mice	8 weeks old	miR-29c exosome injection	Animal model	Skeletal muscle	Increased myofiber cross-sectional area, reduced atrophy factors	Dual regulation by miR-29c: decreased TRIM63 (MuRF1)/FBXO32 (atrogin-1); enhanced satellite cell proliferation and differentiation
Li et al. (2025b)	Tendinopathy	Not reported	Tendon stem cell exosomes + ECM hydrogel	Animal model	Tendon tissue	Enhanced tendon regeneration, reduced inflammatory infiltration	Promoted M2 macrophage polarization; decreased TNF-α/IL-1β
Chen et al. (2018)	Osteoarthritis	Not reported	IL-1β-pretreated HucMSCs exosomes	Cell model	Chondrocytes/macrophages	Increased cartilage matrix production, promoted M2 macrophage polarization	miRNA regulated inflammatory pathways; decreased pro-inflammatory factors, improved anti-inflammatory microenvironment

Abbreviations: *Ev*, Extracellular Vesicles; *FGF21*, Fibroblast Growth Factor 21; *ANGPTL4*, Angiopoietin-like Protein 4; *LPL*, lipoprotein lipase; *miR-122-5p*, MicroRNA-122-5p; *Nrf2*, Nuclear Factor Erythroid 2; *CPT1a*, Carnitine Palmitoyltransferase 1a; *IGF-1*, Insulin-like Growth Factor 1; *PTEN*, phosphatase and tensin homolog; *PEDF*, Pigment Epithelium-Derived Factor; *IL-8*, Interleukin-8; *mRNA*, Messenger RNA; *HSP60*, Heat Shock Protein 60; *BMI*, body mass index; *miR-29c*, MicroRNA-29c; *TNF-α*, Tumor Necrosis Factor-α; *IL-1β*, Interleukin-1β; *HucMSCs*, Human Umbilical Cord Mesenchymal Stem Cells; *ECM*, extracellular matrix.

Exercise can counteract lipotoxicity by improving lipid turnover and lipid droplet quality. Within the pathological context of sarcopenia, aging muscle frequently exhibits disordered lipid metabolism and ectopic lipid deposition, which exacerbates insulin resistance and muscle loss; therefore, exercise intervention targeting lipotoxicity is of significant importance for delaying sarcopenia. In a review focusing on the latest human data, it was found that lipotoxicity is typically characterized by an increase in bioactive lipid species such as ceramides. For example, in obese individuals, exercise training reduces intramuscular ceramide content without necessarily decreasing ectopic lipid storage. Evidence also indicates that exercise training elevates markers of lipid droplet dynamics, including PLIN proteins, triglyceride lipases ATGL and HSL, as well as mitochondrial efficiency. This may explain the improved lipid turnover and reduced accumulation of lipotoxic intermediate products observed in athletes, despite their increased intramuscular lipid levels (Zacharewicz et al., 2018). These findings suggest that exercise likely protects aged muscle from lipotoxic damage by optimizing the balance between lipid storage and utilization, thereby helping to preserve muscle mass and function-offering a mechanistic rationale for exercise in the prevention and management of sarcopenia.

In a study on the cardioprotective mechanism of Turbo cornutus, oxidative damage was induced in freshly isolated hearts by incubation with 0.1 mM FeSO_4_ *in vitro*. Treatments were performed by co-incubation with Turbo cornutus extract or gallic acid (as a standard antioxidant). The induction of oxidative cardiac damage led to significant changes in lipid-related parameters, such as depletion of glutathione, triglycerides, HDL-cholesterol, superoxide, catalase, and ENTPDase activity levels, while increasing malondialdehyde, cholesterol, LDL-cholesterol, ACE, acetylcholinesterase, ATPase, and lipase activity levels. These levels and activities were significantly reversed after Turbo cornutus treatment, indicating its ability to alleviate lipotoxicity and regulate dysregulated cardiac metabolic activities (Erukainure et al., 2021). Although this study focused on cardiac tissue, its demonstrated mechanisms of modulating lipid metabolism and oxidative stress provide a valuable reference for understanding how exercise-derived exosomes might mitigate lipotoxicity in aged muscle. In sarcopenia, exercise may, through the induced release of exosomes with analogous regulatory functions, indirectly help improve the lipid metabolic state of muscle, thereby counteracting age-related muscle loss.

### 3.2 Maintaining muscle protein homeostasis

Exercise-derived exosomes carrying miR-29c play a key role in maintaining muscle protein homeostasis, with critical importance for counteracting the pathological core of net protein loss in sarcopenia (Silva et al., 2019). Through a dual regulatory mechanism, miR-29c coordinately balances muscle protein synthesis and catabolism-directly addressing the two major defects in sarcopenia: excessive catabolism and insufficient anabolism (Silva et al., 2019; Alves et al., 2022; Xie et al., 2021). On one hand, miR-29c directly inhibits the expression of muscle-atrophy-related ubiquitin ligases TRIM63/MuRF1 and FBXO32/atrogin-1, thereby reducing muscle protein degradation. On the other hand, it promotes the proliferation and differentiation of satellite cells, enhancing muscle protein synthesis. During aging, the decline in satellite-cell function and the exacerbation of anabolic resistance make this dual regulation particularly crucial in the context of sarcopenia (Silva et al., 2019; Li J. et al., 2017).

This dual action has been validated in cellular and animal models: in C2C12 myoblast experiments, miR-29c oligonucleotide treatment enhanced cell differentiation, as reflected by increased immunostaining of myocyte enhancer factor-2 (MEF2), elevated myotube fusion index, and upregulated mRNA levels of myogenesis-related markers (Wu et al., 2025). *In vivo*, overexpression of miR-29c increased the mass of the tibialis anterior muscle, accompanied by greater fiber cross-sectional area and strength (Wu et al., 2025). Notably, in a mouse model of muscle atrophy induced by unilateral ureteral obstruction (UUO), injection of exosomes carrying miR-29c increased myofiber cross-sectional area and reduced the expression of the aforementioned atrophy-related factors, thereby ameliorating muscle wasting (Wang et al., 2019). These results not only confirm the function of miR-29c but also suggest the translational potential of exosome-based delivery systems in treating age-related muscle atrophy.

Studies on other miRNA family members provide context and reference for understanding the regulatory network of miR-29c. For example, miR-34b has been shown to promote muscle growth and development *in vivo* by targeting SYISL and regulating its downstream genes p21 and MyoG (Wu et al., 2025). In addition, angiotensin II-induced muscle atrophy via PPARγ suppression is mediated by miR-29b; PPARγ acts as a negative regulator of miR-29b, and inhibition of miR-29b prevents such atrophy. IGF1, PI3K (p85α), and YY1 have been identified as target genes of miR-29b (Li et al., 2021). These findings reveal the important role of miR-29/34 family members in muscle homeostasis. Collectively, studies in aging or stress models outline a key network of miRNAs in the regulation of muscle mass, providing an evolutionary and mechanistic context for understanding the function of miR-29c in sarcopenia. Although the mechanistic insights from these related miRNAs are instructive, miR-29c in exercise-derived exosomes likely plays a central role in maintaining muscle protein homeostasis through its own unique, exercise-stress-adapted regulatory network. This suggests that promoting the release of miR-29c-enriched exosomes via exercise, or developing them as biologics, could become an innovative strategy to improve muscle protein metabolic imbalance in patients with sarcopenia. Future studies should focus on whether exercise-induced exosomal miR-29c signaling is sufficient to counteract sarcopenia progression in aged individuals and how this pathway can be optimized for better clinical outcomes.

### 3.3 Regulating the inflammatory microenvironment

Extracellular vesicles play a crucial role in regulating the microenvironment of muscle inflammation, and this process is essential for improving chronic low-grade inflammation and reversing muscle regeneration disorders in sarcopenia. The dysregulation of the inflammatory microenvironment is one of the key factors hindering effective muscle regeneration in the process of aging and sarcopenia. After muscle injury, exosomes can modulate the polarization state of macrophages, promoting their shift from the pro-inflammatory M1 phenotype to the anti-inflammatory M2 phenotype. Exosomes enriched with miR-125b-5p effectively induce M2 macrophage polarization, thereby supporting the activation of muscle stem cells and muscle repair (Li D. et al., 2025; Chazaud, 2020; Yang et al., 2025). The immune regulatory mechanism mediated by this extracellular vesicle is of great significance for correcting the chronic inflammatory state that persists in aging muscles and is not conducive to regeneration.

In the context of chronic inflammation in sarcopenia, this exosome mediated immune regulation is expected to break the pro-inflammatory cycle. The exosomes derived from human umbilical cord mesenchymal stem cells (HucMSCs) pretreated with interleukin-1β (IL-1β) exhibit enhanced anti-inflammatory properties. Exosomes secreted by fibroadipogenic progenitors (FAPs) can promote the activation of muscle stem cells and muscle regeneration by carrying specific microRNAs (Yu et al., 2024), among which miR-206 regulates the deposition of muscle extracellular matrix and the remodeling of muscle tissue (Spinazzola and Gussoni, 2017), improve the inflammatory microenvironment that is unfavorable for regeneration, thereby promoting muscle cell regeneration (Chen et al., 2025).

This suggests that targeting the inflammatory microenvironment may be an important strategy for intervening in sarcopenia. In addition to regulating the proliferation and differentiation of muscle stem cells, exosomes can directly promote the formation and regeneration of muscle fibers by carrying growth factors and other signaling molecules (Luo et al., 2024; Porcu et al., 2024). Exosomes derived from human skeletal muscle cells can induce adipocyte-derived stem cells to undergo myogenesis and significantly improve muscle regeneration in muscle injury models (Luo et al., 2024). These findings collectively indicate that extracellular vesicles play an indispensable role in regulating the inflammatory microenvironment of muscles and promoting regeneration through multiple mechanisms, providing new ideas for intervening in sarcopenia characterized by inflammation and regeneration defects. Future research can further explore the potential of extracellular vesicles in the treatment of muscle diseases, especially for sarcopenia, an aging related disease, and develop extracellular therapeutic strategies based on extracellular vesicles (Wan et al., 2022; Cobelli et al., 2017).

## 4 Exosomes: an innovative direction for sarcopenia treatment

In the context of aging and sarcopenia, identifying effective strategies to intervene in regenerative impairment is crucial. The maintenance and decline of skeletal muscle regenerative capacity represent central issues in elucidating the pathogenesis of sarcopenia and developing intervention strategies. Owing to their unique biological properties, exosomes are emerging as a highly promising research direction in this field. The self-regeneration of skeletal muscle primarily relies on the fusion of satellite cells with damaged muscle fibers to achieve repair and remodeling (Bi et al., 2018). However, during the progression of aging and sarcopenia, satellite cell numbers decrease significantly, their function declines markedly, and the distance between satellite cells and capillaries increases, leading to dysregulated transmission of activation signals and further impairing muscle remodeling and regenerative capacity (Joanisse et al., 2017). Notably, sarcopenia exerts a more pronounced impact on fast-twitch muscle fibers in the elderly. Compared to corresponding slow-twitch fibers, these fibers exhibit lower expression levels of myosin, actin chaperones, and proteasome activity, which exacerbates functional loss and complicates repair in aged muscle (Murgia et al., 2017; Purves-Smith et al., 2014; Larsson et al., 2019).

In this context, the role of exosomes in the regulation of aged muscle regeneration is becoming increasingly prominent: exosomes can carry non-coding RNA molecules such as miRNAs, participating in the fine regulation of skeletal muscle regeneration. miRNAs are involved in post-transcriptional gene regulation through mRNA degradation or translational inhibition. In aged muscle, the dysregulated expression of multiple key miRNAs is associated with regenerative deficits. Skeletal muscle is rich in functionally specific miRNAs, such as miR-1, miR-133, miR-206, and miR-486 (Hausser and Zavolan, 2014; Zhang et al., 2014; Wang et al., 2012; Iwakawa and Tomari, 2015). The expression levels of miR-133b and miR-181a-5p are significantly upregulated in exercise-induced exosomes, which may contribute to muscle remodeling and homeostasis maintenance by modulating satellite cell activation and muscle regeneration-related pathways (Guescini et al., 2015); miR-31 can regulate the translation of the satellite cell activator Myf5, directly influencing the muscle regeneration process (Lovet et al., 2018; Crist et al., 2012); miR-23a/27a can also reduce myofiber loss by inhibiting muscle atrophy-related genes (Zhang et al., 2018).

Most of these miRNAs are secreted via extracellular vesicles such as exosomes and delivered to target cells, forming a trans-cellular regulatory network (Sun et al., 2018). Of particular note is that aging may alter the miRNA cargo profile of exosomes. Conversely, if muscle-derived exosomes carry aging-associated miRNAs (e.g., miR-34a-5p), they may induce senescence in skeletal stem cells and exacerbate regenerative impairment (Fulzele et al., 2019). This reveals a potential shift in exosome function from pro-regenerative to pro-senescent during aging, offering specific molecular targets for therapeutic intervention. Exosomes possess strong signal-transduction capabilities. Exosomes derived from human skeletal muscle cells can trigger the myogenic differentiation of stem cells, providing important biochemical cues for muscle regeneration. The myogenic growth factors they carry likely play a central role in this process (Choi et al., 2016). Meanwhile, a study by Mobley et al. was the first to demonstrate that whey-protein-derived exosomes can increase muscle protein synthesis and hypertrophy *in vitro*, possibly by up-regulating translation initiation factors. This mechanism operates independently of the mTOR signaling pathway, suggesting a distinct mode of anabolic regulation (Mobley et al., 2017). These findings provide a theoretical basis for using exogenous or engineered exosomes to bypass intracellular signaling defects in aged cells and directly stimulate anabolism.

Exosomes can be engineered into targeted delivery systems carrying specific DNA, RNA, proteins, or drugs. This property has demonstrated research value in various disease models, such as sarcopenia, type 2 diabetes, hind-limb ischemia, temporomandibular joint osteoarthritis, sepsis-induced kidney injury, myocardial infarction, Parkinson’s disease, and gastric cancer (Nam et al., 2020; Le Bras, 2018; Tran et al., 2020). Applying this property to aging-related sarcopenia research would enable precise modulation of core pathological processes-including satellite-cell dysfunction, inflammatory microenvironment disturbance, and protein-metabolic imbalance-offering broad prospects for developing individualized intervention strategies. By regulating satellite-cell function, balancing miRNA regulatory networks, mediating intercellular signal transmission, and achieving targeted delivery, exosomes directly participate in key steps of skeletal muscle regeneration. They exhibit unique potential in reversing or delaying aging-related regenerative decline, undoubtedly providing a new perspective and a potential breakthrough for research on skeletal muscle regeneration mechanisms and the development of sarcopenia prevention and treatment strategies.

A central and pressing translational question concerns the interplay between exercise intensity, age, and the release and function of exosomes. The release of exosomes exhibits a marked intensity-dependence. High-intensity exercise can more rapidly and substantially increase the number of circulating exosomes (Deng et al., 2021; Wa et al., 2025). However, older adults with sarcopenia are often unable to safely tolerate high-intensity training due to comorbidities, musculoskeletal risk, or functional limitations (Liu et al., 2022). Therefore, moderate-to low-intensity exercise becomes a more feasible alternative. Unlike the exosome profile enriched in “potent pro-regenerative” signals observed after high-intensity exercise in younger individuals, exosomes induced by moderate-to low-intensity exercise in older adults may carry a cargo biased toward molecules characterized as “protective,” “adaptive,” and “microenvironment-optimizing.” These may include factors that improve vascular function and perfusion, molecules that effectively suppress chronic inflammation, effectors that enhance autophagy and cellular clearance, and signals that promote metabolic flexibility (Lai et al., 2023; Wang et al., 2020).

Such exosomes may partly compensate for the reduced “potent pro-regenerative” signals resulting from limited exercise intensity by optimizing the inflammatory and fibrotic microenvironment of aged muscle, enhancing the sensitivity of residual satellite cells to limited mitogenic signals, and improving overall tissue metabolic health-thereby supporting the maintenance and repair of aged muscle (Li et al., 2019; Sun et al., 2025; Liu et al., 2024). Future studies urgently need to directly compare the molecular profiles of exosomes released by individuals of different ages under different exercise intensities. This will clarify how aging alters the exercise–exosome response and identify the most effective “exercise–exosome” signatures for improving sarcopenia in the older population, laying the groundwork for developing precise exercise prescriptions or exosome-based alternative therapies.

## 5 Conclusion

The pathological core of sarcopenia lies in the synergistic interplay of imbalanced protein synthesis and degradation, autophagy-mitochondrial axis dysfunction, chronic low-grade inflammation, and the functional decline of muscle stem cells coupled with microenvironmental dysregulation, which collectively drive the progressive loss of skeletal muscle mass and function. As a safe and accessible non-pharmacological intervention, exercise integrates multiple physiological stimuli-including mechanical stress, calcium signaling, metabolic stress, and neuroendocrine regulation—to markedly enhance the release of circulating exosomes derived from diverse tissue sources. These exercise-induced exosomes carry a rich cargo of proteins, lipids, and nucleic acids, forming a systemic intercellular communication network. This review systematically elucidates the multi-layered reparative mechanisms by which exercise-derived exosomes counteract sarcopenia: alleviating muscle lipotoxicity through pathways such as activation of the FGF21-adiponectin axis; dually regulating protein metabolism via key molecules like miR-29c, which suppresses ubiquitin–proteasome-mediated catabolism while promoting satellite cell-driven anabolism; and improving the chronic inflammatory microenvironment by modulating macrophage polarization, thereby creating a favorable milieu for muscle regeneration. Together, these mechanisms directly target the core pathological processes of sarcopenia, reflecting the role of exosomes as a molecular bridge conveying the systemic benefits of exercise to local tissue repair.

Exercise-derived exosomes represent a highly promising novel target for the prevention and management of sarcopenia. Future research should aim to: clarify the specific regulatory effects of different exercise modalities on exosomal secretion profiles and molecular cargo; validate the therapeutic efficacy of specific exosome subpopulations in aging and sarcopenia animal models, while exploring engineering strategies to enhance their targeting and functional specificity; and conduct clinical studies to characterize exosome responses to exercise in older adults with varying health statuses, thereby laying a translational foundation for developing personalized exercise prescriptions or exosome-based biologics.

## References

[B1] Agarwal A. Fanelli G. Letizia M. Tung S. L. Boardman D. Lechler R. (2014). Regulatory T cell-derived exosomes: possible therapeutic and diagnostic tools in transplantation. Front. Immunol. 5, 555. 10.3389/fimmu.2014.00555 25414702 PMC4220709

[B2] Akkız H. Şimşek H. Balcı D. Ülger Y. Onan E. Akçaer N. (2025). Inflammation and cancer: molecular mechanisms and clinical consequences. Front. Oncol. 15, 1564572. 10.3389/fonc.2025.1564572 40165901 PMC11955699

[B3] Alonso-Puyo J. Izagirre-Fernandez O. Crende O. Valdivia A. García-Gallastegui P. Sanz B. (2024). Experimental models as a tool for research on sarcopenia: a narrative review. Ageing Res. Rev. 101, 102534. 10.1016/j.arr.2024.102534 39369798

[B4] Alves P. K. N. Cruz A. Silva W. J. Labeit S. Moriscot A. S. (2022). miR-29c increases protein synthesis in skeletal muscle independently of AKT/mTOR. Int. J. Mol. Sci. 23, 2313. 10.3390/ijms23137198 35806204 PMC9266809

[B5] Amemiya Y. Nakamura N. Ikeda N. Sugiyama R. Ishii C. Maki M. (2021). Amino acid-mediated intracellular Ca(2+) rise modulates mTORC1 by regulating the TSC2-Rheb axis through Ca(2+)/Calmodulin. Int. J. Mol. Sci. 22, 2213. 10.3390/ijms22136897 34198993 PMC8269083

[B6] Annibalini G. Contarelli S. Lucertini F. Guescini M. Maggio S. Ceccaroli P. (2019). Muscle and systemic molecular responses to a single flywheel based iso-inertial training session in resistance-trained men. Front. Physiol. 10, 554. 10.3389/fphys.2019.00554 31143128 PMC6521220

[B7] Arnold L. Henry A. Poron F. Baba-Amer Y. van Rooijen N. Plonquet A. (2007). Inflammatory monocytes recruited after skeletal muscle injury switch into antiinflammatory macrophages to support myogenesis. J. Exp. Med. 2045, 1057–1069. 10.1084/jem.20070075 17485518 PMC2118577

[B8] Barone R. Macaluso F. Sangiorgi C. Campanella C. Marino Gammazza A. Moresi V. (2016). Skeletal muscle heat shock protein 60 increases after endurance training and induces peroxisome proliferator-activated receptor gamma coactivator 1 α1 expression. Sci. Rep. 6, 19781. 10.1038/srep19781 26812922 PMC4728392

[B9] Bi P. McAnally J. R. Shelton J. M. Sánchez-Ortiz E. Bassel-Duby R. Olson E. N. (2018). Fusogenic micropeptide myomixer is essential for satellite cell fusion and muscle regeneration. Proc. Natl. Acad. Sci. U. S. A. 11515, 3864–3869. 10.1073/pnas.1800052115 29581287 PMC5899482

[B10] Bigot A. Duddy W. J. Ouandaogo Z. G. Negroni E. Mariot V. Ghimbovschi S. (2015). Age-Associated methylation suppresses SPRY1, leading to a failure of Re-quiescence and loss of the reserve Stem cell pool in elderly muscle. Cell Rep. 136, 1172–1182. 10.1016/j.celrep.2015.09.067 26526994

[B11] Bissig C. Gruenberg J. (2014). ALIX and the multivesicular endosome: ALIX in Wonderland. Trends Cell Biol. 241, 19–25. 10.1016/j.tcb.2013.10.009 24287454

[B12] Bocchetti M. Luce A. Iannarone C. Pasquale L. S. Falco M. Tammaro C. (2024). Exosomes multiplex profiling, a promising strategy for early diagnosis of laryngeal cancer. J. Transl. Med. 221, 582. 10.1186/s12967-024-05396-0 38902710 PMC11188179

[B13] Borén J. Taskinen M. R. Olofsson S. O. Levin M. (2013). Ectopic lipid storage and insulin resistance: a harmful relationship. J. Intern. Med. 2741, 25–40. 10.1111/joim.12071 23551521

[B14] Bosma M. Kersten S. Hesselink M. K. Schrauwen P. (2012). Re-evaluating lipotoxic triggers in skeletal muscle: relating intramyocellular lipid metabolism to insulin sensitivity. Prog. Lipid Res. 511, 36–49. 10.1016/j.plipres.2011.11.003 22120643

[B15] Bowen T. S. Schuler G. Adams V. (2015). Skeletal muscle wasting in cachexia and sarcopenia: molecular pathophysiology and impact of exercise training. J. Cachexia Sarcopenia Muscle 63, 197–207. 10.1002/jcsm.12043 26401465 PMC4575550

[B16] Brahmer A. Neuberger E. Esch-Heisser L. Haller N. Jorgensen M. M. Baek R. (2019). Platelets, endothelial cells and leukocytes contribute to the exercise-triggered release of extracellular vesicles into the circulation. J. Extracell. Vesicles 81, 1615820. 10.1080/20013078.2019.1615820 31191831 PMC6542154

[B17] Burrello J. Burrello A. Vacchi E. Bianco G. Caporali E. Amongero M. (2022). Supervised and unsupervised learning to define the cardiovascular risk of patients according to an extracellular vesicle molecular signature. Transl. Res. 244, 114–125. 10.1016/j.trsl.2022.02.005 35202881

[B18] Cai Z. Liu D. Yang Y. Xie W. He M. Yu D. (2022). The role and therapeutic potential of stem cells in skeletal muscle in sarcopenia. Stem Cell Res. Ther. 131, 28. 10.1186/s13287-022-02706-5 35073997 PMC8785537

[B19] Castaño C. Mirasierra M. Vallejo M. Novials A. Párrizas M. (2020). Delivery of muscle-derived exosomal miRNAs induced by HIIT improves insulin sensitivity through down-regulation of hepatic FoxO1 in mice. Proc. Natl. Acad. Sci. U. S. A. 11748, 30335–30343. 10.1073/pnas.2016112117 33199621 PMC7720135

[B20] Chakkalakal J. V. Jones K. M. Basson M. A. Brack A. S. (2012). The aged niche disrupts muscle stem cell quiescence. Nature 490, 35–60. 10.1038/nature11438 23023126 PMC3605795

[B21] Chazaud B. (2020). Inflammation and skeletal muscle regeneration: leave it to the macrophages. Trends Immunol. 416, 481–492. 10.1016/j.it.2020.04.006 32362490

[B22] Chen Z. Tao S. Li X. Yao Q. (2018). Resistin destroys mitochondrial biogenesis by inhibiting the PGC-1α/NRF1/TFAM signaling pathway. Biochem. Biophys. Res. Commun. 5041, 13–18. 10.1016/j.bbrc.2018.08.027 30172371

[B23] Chen M. Liu Y. Cao Y. Zhao C. Liu Q. Li N. (2025). Remodeling the proinflammatory microenvironment in osteoarthritis through Interleukin-1 beta tailored exosome cargo for inflammatory regulation and cartilage regeneration. ACS Nano 194, 4924–4941. 10.1021/acsnano.4c16785 39848926

[B24] Chinvattanachot G. Rivas D. Duque G. (2024). Mechanisms of muscle cells alterations and regeneration decline during aging. Ageing Res. Rev. 102, 102589. 10.1016/j.arr.2024.102589 39566742

[B25] Choi J. S. Yoon H. I. Lee K. S. Choi Y. C. Yang S. H. Kim I. S. (2016). Exosomes from differentiating human skeletal muscle cells trigger myogenesis of stem cells and provide biochemical cues for skeletal muscle regeneration. J. Control Release 222, 107–115. 10.1016/j.jconrel.2015.12.018 26699421

[B26] Cobelli N. J. Leong D. J. Sun H. B. (2017). Exosomes: biology, therapeutic potential, and emerging role in musculoskeletal repair and regeneration. Ann. N. Y. Acad. Sci. 14101, 57–67. 10.1111/nyas.13469 29125180

[B27] Colombo M. Raposo G. Théry C. (2014). Biogenesis, secretion, and intercellular interactions of exosomes and other extracellular vesicles. Annu. Rev. Cell Dev. Biol. 30, 255–289. 10.1146/annurev-cellbio-101512-122326 25288114

[B28] Cosgrove B. D. Gilbert P. M. Porpiglia E. Mourkioti F. Lee S. P. Corbel S. Y. (2014). Rejuvenation of the muscle stem cell population restores strength to injured aged muscles. Nat. Med. 203, 255–264. 10.1038/nm.3464 24531378 PMC3949152

[B29] Crist C. G. Montarras D. Buckingham M. (2012). Muscle satellite cells are primed for myogenesis but maintain quiescence with sequestration of Myf5 mRNA targeted by microRNA-31 in mRNP granules. Cell Stem Cell 111, 118–126. 10.1016/j.stem.2012.03.011 22770245

[B30] Cruz-Jentoft A. J. Sayer A. A. (2019). Sarcopenia. Lancet. 393, 26–46. 10.1016/s0140-6736(19)31138-9 31171417

[B31] Cunha E. R. K. Ying W. Olefsky J. M. (2024). Exosome-Mediated impact on systemic metabolism. Annu. Rev. Physiol. 86, 225–253. 10.1146/annurev-physiol-042222-024535 38345906

[B32] D'Souza R. F. Woodhead J. S. T. Zeng N. Blenkiron C. Merry T. L. Cameron-Smith D. (2018). Circulatory exosomal miRNA following intense exercise is unrelated to muscle and plasma miRNA abundances. Am. J. Physiol. Endocrinol. Metab. 3154, E23–e33. 10.1152/ajpendo.00138.2018 29969318

[B33] Deng C. C. Hu Y. F. Zhu D. H. Cheng Q. Gu J. J. Feng Q. L. (2021). Single-cell RNA-seq reveals fibroblast heterogeneity and increased mesenchymal fibroblasts in human fibrotic skin diseases. Nat. Commun. 121, 3709. 10.1038/s41467-021-24110-y 34140509 PMC8211847

[B34] Drummond M. J. Dickinson J. M. Fry C. S. Walker D. K. Gundermann D. M. Reidy P. T. (2012). Bed rest impairs skeletal muscle amino acid transporter expression, mTORC1 signaling, and protein synthesis in response to essential amino acids in older adults. Am. J. Physiol. Endocrinol. Metab. 3029, E1113–E1122. 10.1152/ajpendo.00603.2011 22338078 PMC3361979

[B35] Durham W. J. Dillon E. L. Sheffield-Moore M. (2009). Inflammatory burden and amino acid metabolism in cancer cachexia. Curr. Opin. Clin. Nutr. Metab. Care 121, 72–77. 10.1097/MCO.0b013e32831cef61 19057191 PMC2742684

[B36] Erukainure O. L. Chukwuma C. I. Matsabisa M. G. Javu M. T. Salau V. F. Koorbanally N. A. (2021). Turbina oblongata protects against oxidative cardiotoxicity by suppressing lipid dysmetabolism and modulating cardiometabolic activities linked to Cardiac dysfunctions. Front. Pharmacol. 12, 610835. 10.3389/fphar.2021.610835 34093172 PMC8174711

[B37] Fader C. M. Sánchez D. G. Mestre M. B. Colombo M. I. (2009). TI-VAMP/VAMP7 and VAMP3/cellubrevin: two v-SNARE proteins involved in specific steps of the autophagy/multivesicular body pathways. Biochim. Biophys. Acta 1793, 1–16. 10.1016/j.bbamcr.2009.09.011 19781582

[B38] Fan Z. Turiel G. Ardicoglu R. Ghobrial M. Masschelein E. Kocijan T. (2021). Exercise-induced angiogenesis is dependent on metabolically primed ATF3/4(+) endothelial cells. Cell Metab. 339, 1793–1807.e9. 10.1016/j.cmet.2021.07.015 34358431 PMC8432967

[B39] Fan Y. Pionneau C. Cocozza F. Boëlle P. Y. Chardonnet S. Charrin S. (2023). Differential proteomics argues against a general role for CD9, CD81 or CD63 in the sorting of proteins into extracellular vesicles. J. Extracell. Vesicles 128, e12352. 10.1002/jev2.12352 37525398 PMC10390663

[B40] Feng L. T. Chen Z. N. Bian H. (2020). Skeletal muscle: molecular structure, myogenesis, biological functions, and diseases. MedComm 5, 57:e649. 10.1002/mco2.649 38988494 PMC11234433

[B41] Feng Z. Xia J. Yu J. Wang J. Yin S. Yang J. (2025). Pathophysiological mechanisms underlying sarcopenia and sarcopenic obesity: a systematic review and meta-analysis of biomarker evidence. Int. J. Mol. Sci. 26, 2611. 10.3390/ijms26115113 40507924 PMC12154750

[B42] Frühbeis C. Helmig S. Tug S. Simon P. Krämer-Albers E. M. (2015). Physical exercise induces rapid release of small extracellular vesicles into the circulation. J. Extracell. Vesicles 4, 28239. 10.3402/jev.v4.28239 26142461 PMC4491306

[B43] Fry C. S. Lee J. D. Mula J. Kirby T. J. Jackson J. R. Liu F. (2015). Inducible depletion of satellite cells in adult, sedentary mice impairs muscle regenerative capacity without affecting sarcopenia. Nat. Med. 211, 76–80. 10.1038/nm.3710 25501907 PMC4289085

[B44] Fu X. Xiao J. Wei Y. Li S. Liu Y. Yin J. (2015). Combination of inflammation-related cytokines promotes long-term muscle stem cell expansion. Cell Res. 259, 1–3. 10.1038/cr.2015.102 26323492 PMC4559808

[B45] Fulzele S. Mendhe B. Khayrullin A. Johnson M. Kaiser H. Liu Y. (2019). Muscle-derived miR-34a increases with age in circulating extracellular vesicles and induces senescence of bone marrow stem cells. Aging (Albany NY) 116, 1791–1803. 10.18632/aging.101874 30910993 PMC6461183

[B46] Geng Z. Sun T. Yu J. Wang N. Jiang Q. Wang P. (2024). Cinobufagin suppresses lipid peroxidation and inflammation in osteoporotic mice by promoting the delivery of miR-3102-5p by macrophage-derived exosomes. Int. J. Nanomedicine 19, 10497–10512. 10.2147/ijn.S483849 39439501 PMC11495194

[B47] Gomes C. P. Oliveira G. P. Jr. Madrid B. Almeida J. A. Franco O. L. Pereira R. W. (2014). Circulating miR-1, miR-133a, and miR-206 levels are increased after a half-marathon run. Biomarkers 197, 585–589. 10.3109/1354750x.2014.952663 25146754

[B48] Gomes J. L. Fernandes T. Soci U. P. Silveira A. C. Barretti D. L. Negrão C. E. (2017). Obesity downregulates MicroRNA-126 inducing capillary rarefaction in skeletal muscle: effects of aerobic exercise training. Oxid. Med. Cell Longev. 2017, 2415246. 10.1155/2017/2415246 28367267 PMC5358469

[B49] Goode A. Butler K. Long J. Cavey J. Scott D. Shaw B. (2016). Defective recognition of LC3B by mutant SQSTM1/p62 implicates impairment of autophagy as a pathogenic mechanism in ALS-FTLD. Autophagy 127, 1094–1104. 10.1080/15548627.2016.1170257 27158844 PMC4990988

[B50] Gross J. C. Chaudhary V. Bartscherer K. Boutros M. (2012). Active Wnt proteins are secreted on exosomes. Nat. Cell Biol. 1410, 1036–1045. 10.1038/ncb2574 22983114

[B51] Guescini M. Canonico B. Lucertini F. Maggio S. Annibalini G. Barbieri E. (2015). Muscle releases alpha-sarcoglycan positive extracellular vesicles carrying miRNAs in the bloodstream. PLoS One 105, e0125094. 10.1371/journal.pone.0125094 25955720 PMC4425492

[B52] Guillon E. Bacar H. Gilquin L. Sasaki T. Mourikis P. Ruggiero F. (2025). Unraveling the matrisome signatures of quiescent and activated muscle stem cells. Stem Cell Rep. 20, 2010. 10.1016/j.stemcr.2025.102635 40939595 PMC12790712

[B53] Hsu C. Morohashi Y. Yoshimura S. Manrique-Hoyos N. Jung S. Lauterbach M. A. (2010). Regulation of exosome secretion by Rab35 and its GTPase-activating proteins TBC1D10A-C. J. Cell Biol. 1892, 223–232. 10.1083/jcb.200911018 20404108 PMC2856897

[B54] Hausser J. Zavolan M. (2014). Identification and consequences of miRNA-target interactions--beyond repression of gene expression. Nat. Rev. Genet. 159, 599–612. 10.1038/nrg3765 25022902

[B55] He R. Peng J. Yuan P. Xu F. Wei W. (2015). Divergent roles of BECN1 in LC3 lipidation and autophagosomal function. Autophagy 115, 740–747. 10.1080/15548627.2015.1034404 25955014 PMC4509441

[B56] Hoffman N. J. Whitfield J. Xiao D. Radford B. E. Suni V. Blazev R. (2025). Phosphoproteomics uncovers exercise intensity-specific skeletal muscle signaling networks underlying high-intensity interval training in healthy Male participants. Sports Med. 558, 1983–2004. 10.1007/s40279-025-02217-2 40257739 PMC12460488

[B57] Hou Z. Qin X. Hu Y. Zhang X. Li G. Wu J. (2019). Longterm exercise-derived exosomal miR-342-5p: a novel exerkine for cardioprotection. Circ. Res. 1249, 1386–1400. 10.1161/circresaha.118.314635 30879399

[B58] Isaac R. Reis F. C. G. Ying W. Olefsky J. M. (2021). Exosomes as mediators of intercellular crosstalk in metabolism. Cell Metab. 339, 1744–1762. 10.1016/j.cmet.2021.08.006 34496230 PMC8428804

[B59] Iwakawa H. O. Tomari Y. (2015). The functions of MicroRNAs: mRNA decay and translational repression. Trends Cell Biol. 2511, 651–665. 10.1016/j.tcb.2015.07.011 26437588

[B60] Joanisse S. Nederveen J. P. Snijders T. McKay B. R. Parise G. (2017). Skeletal muscle regeneration, repair and remodelling in aging: the importance of muscle stem cells and vascularization. Gerontology 631, 91–100. 10.1159/000450922 27760421

[B61] Jørgensen M. Bæk R. Pedersen S. Søndergaard E. K. Kristensen S. R. Varming K. (2013). Extracellular Vesicle (EV) Array: microarray capturing of exosomes and other extracellular vesicles for multiplexed phenotyping. J. Extracell. Vesicles 2, 2. 10.3402/jev.v2i0.20920 24009888 PMC3760630

[B62] Jung V. Vincent-Cuaz C. Tumescheit C. Fournier L. Darsinou M. Xu Z. M. (2025). Decoding the interactions and functions of non-coding RNA with artificial intelligence. Nat. Rev. Mol. Cell Biol. 2610, 797–818. 10.1038/s41580-025-00857-w 40537558

[B63] Kalluri R. LeBleu V. S. (2020). The biology, function, and biomedical applications of exosomes. Science 367, 3676478. 10.1126/science.aau6977 32029601 PMC7717626

[B64] Karvinen S. Sievänen T. Karppinen J. E. Hautasaari P. Bart G. Samoylenko A. (2020). MicroRNAs in extracellular vesicles in sweat change in response to endurance exercise. Front. Physiol. 11, 676. 10.3389/fphys.2020.00676 32760282 PMC7373804

[B65] Kawanishi N. Tominaga T. Suzuki K. (2023). Electrical pulse stimulation-induced muscle contraction alters the microRNA and mRNA profiles of circulating extracellular vesicles in mice. Am. J. Physiol. Regul. Integr. Comp. Physiol. 3246, R761–r771. 10.1152/ajpregu.00121.2022 37092746

[B66] Kim B. Ronaldo R. Kweon B. N. Yoon S. Park Y. Baek J. H. (2024). Mesenchymal stem cell-derived exosomes attenuate hepatic steatosis and insulin resistance in diet-induced Obese mice by activating the FGF21-Adiponectin axis. Int. J. Mol. Sci. 25, 2519. 10.3390/ijms251910447 39408777 PMC11476820

[B67] King H. W. Michael M. Z. Gleadle J. M. (2012). Hypoxic enhancement of exosome release by breast cancer cells. BMC Cancer 12, 421. 10.1186/1471-2407-12-421 22998595 PMC3488584

[B68] Kob R. Fellner C. Bertsch T. Wittmann A. Mishura D. Sieber C. C. (2015). Gender-specific differences in the development of sarcopenia in the rodent model of the ageing high-fat rat. J. Cachexia Sarcopenia Muscle 62, 181–191. 10.1002/jcsm.12019 26136194 PMC4458084

[B69] Lai Z. Liang J. Zhang J. Mao Y. Zheng X. Shen X. (2023). Exosomes as a delivery tool of exercise-induced beneficial factors for the prevention and treatment of cardiovascular disease: a systematic review and meta-analysis. Front. Physiol. 14, 14. 10.3389/fphys.2023.1190095 37841310 PMC10570527

[B70] Larsson L. Degens H. Li M. Salviati L. Lee Y. I. Thompson W. (2019). Sarcopenia: Aging-Related loss of muscle mass and function. Physiol. Rev. 991, 427–511. 10.1152/physrev.00061.2017 30427277 PMC6442923

[B71] Le Bras A. (2018). Exosome-based therapy to repair the injured heart. Nat. Rev. Cardiol. 157, 382. 10.1038/s41569-018-0027-7 29748593

[B72] Lemos D. R. Babaeijandaghi F. Low M. Chang C. K. Lee S. T. Fiore D. (2015). Nilotinib reduces muscle fibrosis in chronic muscle injury by promoting TNF-mediated apoptosis of fibro/adipogenic progenitors. Nat. Med. 217, 786–794. 10.1038/nm.3869 26053624

[B73] Li J. Hou B. Tumova S. Muraki K. Bruns A. Ludlow M. J. (2014). Piezo1 integration of vascular architecture with physiological force. Nature 515, 2–82. 10.1038/nature13701 25119035 PMC4230887

[B74] Li W. Li C. Zhou T. Liu X. Liu X. Li X. (2017a). Role of exosomal proteins in cancer diagnosis. Mol. Cancer 161, 145. 10.1186/s12943-017-0706-8 28851367 PMC5576100

[B75] Li J. Chan M. C. Yu Y. Bei Y. Chen P. Zhou Q. (2017b). miR-29b contributes to multiple types of muscle atrophy. Nat. Commun. 8, 15201. 10.1038/ncomms15201 28541289 PMC5458521

[B76] Li M. Jiang M. Meng J. Tao L. (2019). Exosomes: carriers of pro-fibrotic signals and therapeutic targets in fibrosis. Curr. Pharm. Des. 2542, 4496–4509. 10.2174/1381612825666191209161443 31814552

[B77] Li J. Yang T. Sha Z. Tang H. Hua X. Wang L. (2021). Angiotensin II-induced muscle atrophy *via* PPARγ suppression is mediated by miR-29b. Mol. Ther. Nucleic Acids 23, 743–756. 10.1016/j.omtn.2020.12.015 33614226 PMC7868689

[B78] Li W. Chen M. Zhang L. (2025a). Muscle stem cell microenvironment and functions in muscle regeneration. Biomolecules 15, 156. 10.3390/biom15060765 40563407 PMC12190381

[B79] Li D. Li S. He S. He H. Yuan G. Ma B. (2025b). Restoring tendon microenvironment in tendinopathy: macrophage modulation and tendon regeneration with injectable tendon hydrogel and tendon-derived stem cells exosomes. Bioact. Mater 47, 152–169. 10.1016/j.bioactmat.2025.01.016 39906648 PMC11791013

[B80] Liu Q. Q. Xie W. Q. Luo Y. X. Li Y. D. Huang W. H. Wu Y. X. (2022). High intensity interval training: a potential method for treating sarcopenia. Clin. Interv. Aging 17, 857–872. 10.2147/cia.S366245 35656091 PMC9152764

[B81] Liu H. Yuan S. Liu G. Li J. Zheng K. Zhang Z. (2024). Satellite cell-derived exosomes: a novel approach to alleviate skeletal muscle atrophy and fibrosis. Adv. Biol. (Weinh) 8, 84. 10.1002/adbi.202300558 38329214

[B82] Lovett J. A. C. Durcan P. J. Myburgh K. H. (2018). Investigation of circulating extracellular vesicle MicroRNA following two consecutive bouts of muscle-damaging exercise. Front. Physiol. 9, 1149. 10.3389/fphys.2018.01149 30177888 PMC6109634

[B83] Luo J. Zhang D. Pu Q. Wen Z. Wu X. Chai J. (2024). Skeletal muscle-derived exosomes selectively coated miRNAs and participate in myoblast proliferation and differentiation mediated by miR-4331-3p. Int. J. Biol. Macromol. 281 (281Pt 1), 136225. 10.1016/j.ijbiomac.2024.136225 39368577

[B84] Lynch C. J. Adams S. H. (2014). Branched-chain amino acids in metabolic signalling and insulin resistance. Nat. Rev. Endocrinol. 1012, 723–736. 10.1038/nrendo.2014.171 25287287 PMC4424797

[B85] Murgia M. Toniolo L. Nagaraj N. Ciciliot S. Vindigni V. Schiaffino S. (2017). Single muscle fiber proteomics reveals fiber-type-specific features of human muscle aging. Cell Rep. 1911, 2396–2409. 10.1016/j.celrep.2017.05.054 28614723

[B86] Meex R. C. R. Blaak E. E. van Loon L. J. C. (2019). Lipotoxicity plays a key role in the development of both insulin resistance and muscle atrophy in patients with type 2 diabetes. Obes. Rev. 209, 1205–1217. 10.1111/obr.12862 31240819 PMC6852205

[B87] Mobley C. B. Mumford P. W. McCarthy J. J. Miller M. E. Young K. C. Martin J. S. (2017). Whey protein-derived exosomes increase protein synthesis and hypertrophy in C(2-)C(12) myotubes. J. Dairy Sci. 1001, 48–64. 10.3168/jds.2016-11341 28341051

[B88] Moore D. R. (2014). Keeping older muscle “young” through dietary protein and physical activity. Adv. Nutr. 55, 599s–607s. 10.3945/an.113.005405 25469405 PMC4188243

[B89] Morley J. E. Anker S. D. von Haehling S. (2014). Prevalence, incidence, and clinical impact of sarcopenia: facts, numbers, and epidemiology-update 2014. J. Cachexia Sarcopenia Muscle 54, 253–259. 10.1007/s13539-014-0161-y 25425503 PMC4248415

[B90] Moro T. Ebert S. M. Adams C. M. Rasmussen B. B. (2016). Amino acid sensing in skeletal muscle. Trends Endocrinol. Metab. 2711, 796–806. 10.1016/j.tem.2016.06.010 27444066 PMC5075248

[B91] Nail H. M. Chiu C. C. Leung C. H. Ahmed M. M. M. Wang H. D. (2023). Exosomal miRNA-mediated intercellular communications and immunomodulatory effects in tumor microenvironments. J. Biomed. Sci. 301, 69. 10.1186/s12929-023-00964-w 37605155 PMC10440907

[B92] Nam G. H. Choi Y. Kim G. B. Kim S. Kim S. A. Kim I. S. (2020). Emerging prospects of exosomes for cancer treatment: from conventional therapy to immunotherapy. Adv. Mater 32, 3251. 10.1002/adma.202002440 33015883

[B93] Obi P. O. Souza T. F. G. Özerkliğ B. Seif S. Bydak B. Klassen N. (2025). Extracellular vesicles released from skeletal muscle post-chronic contractile activity increase mitochondrial biogenesis in recipient myoblasts. J. Extracell. Vesicles 144, e70045. 10.1002/jev2.70045 40205946 PMC11982704

[B94] Ogawa S. Yakabe M. Akishita M. (2016). Age-related sarcopenia and its pathophysiological bases. Inflamm. Regen. 36, 17. 10.1186/s41232-016-0022-5 29259690 PMC5725797

[B95] Ohya T. Miaczynska M. Coskun Ü. Lommer B. Runge A. Drechsel D. (2024). Author Correction: reconstitution of Rab- and SNARE-dependent membrane fusion by synthetic endosomes. Nature 626, E8–e9. 10.1038/s41586-024-07063-2 38273132

[B96] Oliveira G. P. Jr. Porto W. F. Palu C. C. Pereira L. M. Petriz B. Almeida J. A. (2018). Effects of acute aerobic exercise on rats serum extracellular vesicles diameter, concentration and small RNAs content. Front. Physiol. 9, 532. 10.3389/fphys.2018.00532 29881354 PMC5976735

[B97] Ostrowski M. Carmo N. B. Krumeich S. Fanget I. Raposo G. Savina A. (2010). Rab27a and Rab27b control different steps of the exosome secretion pathway. Nat. Cell Biol. 121, 19–30. 10.1038/ncb2000 19966785

[B98] Pegtel D. M. Gould S. J. (2019). Exosomes*. Annu. Rev. Biochem*. 88, 487–514. 10.1146/annurev-biochem-013118-111902 31220978

[B99] Plikus M. V. Wang X. Sinha S. Forte E. Thompson S. M. Herzog E. L. (2021). Fibroblasts: origins, definitions, and functions in health and disease. Cell 18415, 3852–3872. 10.1016/j.cell.2021.06.024 34297930 PMC8566693

[B100] Porcu C. Dobrowolny G. Scicchitano B. M. (2024). Exploring the role of extracellular vesicles in skeletal muscle regeneration. Int. J. Mol. Sci. 25, 2511. 10.3390/ijms25115811 38892005 PMC11171935

[B101] Purves-Smith F. M. Sgarioto N. Hepple R. T. (2014). Fiber typing in aging muscle. Exerc Sport Sci. Rev. 422, 45–52. 10.1249/jes.0000000000000012 24508741

[B102] Rafii S. Butler J. M. Ding B. S. (2016). Angiocrine functions of organ-specific endothelial cells. Nature 529, 16–25. 10.1038/nature17040 26791722 PMC4878406

[B103] Rambacher K. M. Moniri N. H. (2020). The β2-adrenergic receptor-ROS signaling axis: an overlooked component of β2AR function? Biochem. Pharmacol. 171, 113690. 10.1016/j.bcp.2019.113690 31697929 PMC6917825

[B104] Regimbeau M. Abrey J. Vautrot V. Causse S. Gobbo J. Garrido C. (2022). Heat shock proteins and exosomes in cancer theranostics. Semin. Cancer Biol. 86 (86Pt 1), 46–57. 10.1016/j.semcancer.2021.07.014 34343652

[B105] Rigamonti A. E. Bollati V. Pergoli L. Iodice S. De Col A. Tamini S. (2020). Effects of an acute bout of exercise on circulating extracellular vesicles: tissue-sex-and BMI-related differences. Int. J. Obes. (Lond). 445, 1108–1118. 10.1038/s41366-019-0460-7 31578459

[B106] Robbins P. D. Morelli A. E. (2014). Regulation of immune responses by extracellular vesicles. Nat. Rev. Immunol. 143, 195–208. 10.1038/nri3622 24566916 PMC4350779

[B107] Saçma M. Geiger H. (2021). Exercise generates immune cells in bone. Nature 591, 1–2. 10.1038/d41586-021-00419-y 33627859

[B108] Safdar A. Tarnopolsky M. A. (2018). Exosomes as mediators of the systemic adaptations to endurance exercise. Cold Spring Harb. Perspect. Med. 8, 83. 10.1101/cshperspect.a029827 28490541 PMC5830902

[B109] Saikia R. Joseph J. (2021). AMPK: a key regulator of energy stress and calcium-induced autophagy. J. Mol. Med. Berl. 9911, 1539–1551. 10.1007/s00109-021-02125-8 34398293

[B110] Salazar G. Cullen A. Huang J. Zhao Y. Serino A. Hilenski L. (2020). SQSTM1/p62 and PPARGC1A/PGC-1alpha at the interface of autophagy and vascular senescence. Autophagy 166, 1092–1110. 10.1080/15548627.2019.1659612 31441382 PMC7469683

[B111] Savina A. Furlán M. Vidal M. Colombo M. I. (2003). Exosome release is regulated by a calcium-dependent mechanism in K562 cells. J. Biol. Chem. 27822, 20083–20090. 10.1074/jbc.M301642200 12639953

[B112] Savina A. Fader C. M. Damiani M. T. Colombo M. I. (2005). Rab11 promotes docking and fusion of multivesicular bodies in a calcium-dependent manner. Traffic 62, 131–143. 10.1111/j.1600-0854.2004.00257.x 15634213

[B113] Scott D. Blizzard L. Fell J. Jones G. (2011). The epidemiology of sarcopenia in community living older adults: what role does lifestyle play? J. Cachexia Sarcopenia Muscle 23, 125–134. 10.1007/s13539-011-0036-4 21966639 PMC3177044

[B114] Shen Z. Liu C. Liu P. Zhao J. Xu W. (2014). Sphingosine 1-phosphate (S1P) promotes mitochondrial biogenesis in Hep G2 cells by activating Peroxisome proliferator-activated receptor γ coactivator 1α (PGC-1α). Cell Stress Chaperones 194, 541–548. 10.1007/s12192-013-0480-5 24293320 PMC4041936

[B115] Silva W. J. Graça F. A. Cruz A. Silvestre J. G. Labeit S. Miyabara E. H. (2019). miR-29c improves skeletal muscle mass and function throughout myocyte proliferation and differentiation and by repressing atrophy-related genes. Acta Physiol. (Oxf) 226, 2264. 10.1111/apha.13278 30943315 PMC6900115

[B116] Simons M. Raposo G. (2009). Exosomes--vesicular carriers for intercellular communication. Curr. Opin. Cell Biol. 214, 575–581. 10.1016/j.ceb.2009.03.007 19442504

[B117] Singh M. Moniri N. H. (2012). Reactive oxygen species are required for β2 adrenergic receptor-β-arrestin interactions and signaling to ERK1/2. Biochem. Pharmacol. 845, 661–669. 10.1016/j.bcp.2012.06.012 22728070

[B118] Snijders T. Parise G. (2017). Role of muscle stem cells in sarcopenia. Curr. Opin. Clin. Nutr. Metab. Care 203, 186–190. 10.1097/mco.0000000000000360 28376051

[B119] Sousa-Victor P. Muñoz-Cánoves P. (2016). Regenerative decline of stem cells in sarcopenia. Mol. Asp. Med. 50, 109–117. 10.1016/j.mam.2016.02.002 26921790

[B120] Spinazzola J. M. Gussoni E. (2017). Exosomal small talk carries strong messages from muscle stem cells. Cell Stem Cell 201, 1–3. 10.1016/j.stem.2016.12.009 28061348

[B121] Sullivan B. P. Nie Y. Evans S. Kargl C. K. Hettinger Z. R. Garner R. T. (2022). Obesity and exercise training alter inflammatory pathway skeletal muscle small extracellular vesicle microRNAs. Exp. Physiol. 1075, 462–475. 10.1113/ep090062 35293040 PMC9323446

[B122] Sun Z. Shi K. Yang S. Liu J. Zhou Q. Wang G. (2018). Effect of exosomal miRNA on cancer biology and clinical applications. Mol. Cancer 171, 147. 10.1186/s12943-018-0897-7 30309355 PMC6182840

[B123] Sun H. Xia T. Ma S. Lv T. Li Y. (2025). Intercellular communication is crucial in the regulation of healthy aging *via* exosomes. Pharmacol. Res. 212, 107591. 10.1016/j.phrs.2025.107591 39800177

[B124] Théry C. Zitvogel L. Amigorena S. (2002). Exosomes: composition, biogenesis and function. Nat. Rev. Immunol. 28, 569–579. 10.1038/nri855 12154376

[B125] Tomiyama T. Yang G. X. Zhao M. Zhang W. Tanaka H. Wang J. (2017). The modulation of co-stimulatory molecules by circulating exosomes in primary biliary cirrhosis. Cell Mol. Immunol. 143, 276–284. 10.1038/cmi.2015.86 26388238 PMC5360882

[B126] Tran P. H. L. Xiang D. Tran T. T. D. Yin W. Zhang Y. Kong L. (2020). Exosomes and nanoengineering: a match made for precision therapeutics. Adv. Mater 32, 3218. 10.1002/adma.201904040 31531916

[B127] Vechetti I. J. Jr. Peck B. D. Wen Y. Walton R. G. Valentino T. R. Alimov A. P. (2021). Mechanical overload-induced muscle-derived extracellular vesicles promote adipose tissue lipolysis. Faseb J. 356, e21644. 10.1096/fj.202100242R 34033143 PMC8607211

[B128] Wang Z. Ou Y. Zhu X. Zhou Y. Zheng X. Zhang M. (2025). Differential regulation of miRNA and protein profiles in human plasma-derived extracellular vesicles *via* continuous aerobic and high-intensity interval training. Int. J. Mol. Sci. 26, 263. 10.3390/ijms26031383 39941151 PMC11818269

[B129] Wada S. Kato Y. Okutsu M. Miyaki S. Suzuki K. Yan Z. (2011). Translational suppression of atrophic regulators by microRNA-23a integrates resistance to skeletal muscle atrophy. J. Biol. Chem. 28644, 38456–38465. 10.1074/jbc.M111.271270 21926429 PMC3207415

[B130] Wan R. Hussain A. Behfar A. Moran S. L. Zhao C. (2022). The therapeutic potential of exosomes in soft tissue repair and regeneration. Int. J. Mol. Sci. 23, 237. 10.3390/ijms23073869 35409228 PMC8998690

[B131] Wang E. T. Cody N. A. Jog S. Biancolella M. Wang T. T. Treacy D. J. (2012). Transcriptome-wide regulation of pre-mRNA splicing and mRNA localization by muscleblind proteins. Cell 1504, 710–724. 10.1016/j.cell.2012.06.041 22901804 PMC3428802

[B132] Wang H. Wang B. Zhang A. Hassounah F. Seow Y. Wood M. (2019). Exosome-Mediated miR-29 transfer reduces muscle atrophy and kidney fibrosis in mice. Mol. Ther. 273, 571–583. 10.1016/j.ymthe.2019.01.008 30711446 PMC6403486

[B133] Wang J. Liu H. Chen S. Zhang W. Chen Y. Yang Y. (2020). Moderate exercise has beneficial effects on mouse ischemic stroke by enhancing the functions of circulating endothelial progenitor cell-derived exosomes. Exp. Neurol. 330, 113325. 10.1016/j.expneurol.2020.113325 32325158 PMC11055452

[B134] Webster J. M. Kempen L. Hardy R. S. Langen R. C. J. (2020). Inflammation and skeletal muscle wasting during Cachexia. Front. Physiol. 11, 597675. 10.3389/fphys.2020.597675 33329046 PMC7710765

[B135] Wei D. Zhan W. Gao Y. Huang L. Gong R. Wang W. (2021). RAB31 marks and controls an ESCRT-independent exosome pathway. Cell Res. 312, 157–177. 10.1038/s41422-020-00409-1 32958903 PMC8027411

[B136] Wen S. Xu S. Zong X. Wen S. Xiao W. Zheng W. (2025). Association analysis of the circulating proteome with Sarcopenia-Related traits reveals potential drug targets for Sarcopenia. J. Cachexia Sarcopenia Muscle 161, e13720. 10.1002/jcsm.13720 39949133 PMC11825984

[B137] Whitham M. Parker B. L. Friedrichsen M. Hingst J. R. Hjorth M. Hughes W. E. (2018). Extracellular vesicles provide a means for tissue crosstalk during exercise. Cell Metab. 271, 237–251.e4. 10.1016/j.cmet.2017.12.001 29320704

[B138] Whitlock J. M. Hartzell H. C. (2017). Anoctamins/TMEM16 proteins: chloride channels flirting with lipids and extracellular vesicles. Annu. Rev. Physiol. 79, 119–143. 10.1146/annurev-physiol-022516-034031 27860832 PMC5556385

[B139] Wilhelm E. N. González-Alonso J. Parris C. Rakobowchuk M. (2016). Exercise intensity modulates the appearance of circulating microvesicles with proangiogenic potential upon endothelial cells. Am. J. Physiol. Heart Circ. Physiol. 3115, H1297–h1310. 10.1152/ajpheart.00516.2016 27638881

[B140] Wollert T. Hurley J. H. (2010). Molecular mechanism of multivesicular body biogenesis by ESCRT complexes. Nature 464, 8–9. 10.1038/nature08849 20305637 PMC2851844

[B141] Wu Y. Liu X. Fan Y. Zuo H. Niu X. Zuo B. (2025). MiR-34b regulates muscle growth and development by targeting SYISL. Cells 14, 145. 10.3390/cells14050379 40072107 PMC11898696

[B142] Xie K. Xiong H. Xiao W. Xiong Z. Hu W. Ye J. (2021). Downregulation of miR-29c promotes muscle wasting by modulating the activity of leukemia inhibitory factor in lung cancer cachexia. Cancer Cell Int. 211, 627. 10.1186/s12935-021-02332-w 34838029 PMC8626920

[B143] Yanagawa K. Kuma A. Hamasaki M. Kita S. Yamamuro T. Nishino K. (2024). The Rubicon-WIPI axis regulates exosome biogenesis during ageing. Nat. Cell Biol. 269, 1558–1570. 10.1038/s41556-024-01481-0 39174742

[B144] Yang L. Lu R. Cao K. Chen M. Xu X. Cao X. (2024). Regulation of lipid metabolism in grass carp primary hepatocytes by exosomes derived from fatty hepatocytes though GRP78. Fish. Physiol. Biochem. 506, 2287–2299. 10.1007/s10695-024-01384-9 39090453

[B145] Yang M. M. Luo J. Y. Cai J. Qian J. Zhu X. Y. Zou H. Z. (2025). miRNA-125b-5p-rich exosomes derived from fibro-adipogenic progenitors promoting muscle regeneration through inducing pro-regenerative macrophages. Stem Cell Res. Ther. 161, 363. 10.1186/s13287-025-04452-w 40660398 PMC12261530

[B146] Yoshida T. Nakajima H. Takahashi S. Kakizuka A. Imamura H. (2019). OLIVe: a genetically encoded fluorescent biosensor for quantitative imaging of branched-chain amino acid levels inside single living cells. ACS Sens. 412, 3333–3342. 10.1021/acssensors.9b02067 31845569

[B147] Yu Y. Su Y. Wang G. Lan M. Liu J. Garcia M. R. (2024). Reciprocal communication between FAPs and muscle cells *via* distinct extracellular vesicle miRNAs in muscle regeneration. Proc. Natl. Acad. Sci. U. S. A. 121, e2316544121. 10.1073/pnas.2316544121 38442155 PMC10945765

[B148] Zacharewicz E. Hesselink M. K. C. Schrauwen P. (2018). Exercise counteracts lipotoxicity by improving lipid turnover and lipid droplet quality. J. Intern. Med. 2845, 505–518. 10.1111/joim.12729 29331050

[B149] Zhang X. Zuo X. Yang B. Li Z. Xue Y. Zhou Y. (2014). MicroRNA directly enhances mitochondrial translation during muscle differentiation. Cell 1583, 607–619. 10.1016/j.cell.2014.05.047 25083871 PMC4119298

[B150] Zhang A. Li M. Wang B. Klein J. D. Price S. R. Wang X. H. (2018). miRNA-23a/27a attenuates muscle atrophy and renal fibrosis through muscle-kidney crosstalk. J. Cachexia Sarcopenia Muscle 94, 755–770. 10.1002/jcsm.12296 29582582 PMC6104113

[B151] Zhang Y. Su S. A. Li W. Ma Y. Shen J. Wang Y. (2021). Piezo1-Mediated mechanotransduction promotes cardiac hypertrophy by impairing calcium homeostasis to activate Calpain/Calcineurin signaling. Hypertension 783, 647–660. 10.1161/hypertensionaha.121.17177 34333987

[B152] Zylbersztejn K. Galli T. (2011). Vesicular traffic in cell navigation. Febs J. 27823, 4497–4505. 10.1111/j.1742-4658.2011.08168.x 21554543

